# Effects of phytosterol supplementation on lipid profiles and apolipoproteins: A meta-analysis of randomized controlled trials

**DOI:** 10.1097/MD.0000000000040020

**Published:** 2024-10-18

**Authors:** Yi-Feng Zhang, Wanning Qiao, Hanxiao Feng, Kuan Jiang, Jinzhao Yang, Tao Zhou, Yang Zhang

**Affiliations:** a School of Public Health (Shenzhen), Sun Yat-sen University, Shenzhen, Guangdong, China; b Guangdong Provincial Key Laboratory of Diabetology, Guangzhou Key Laboratory of Mechanistic and Translational Obesity Research, The Third Affiliated Hospital of Sun Yat-sen University, Guangzhou, Guangdong, China.

**Keywords:** apolipoprotein, dyslipidemia, lipid profiles, phytosterols

## Abstract

**Background:**

The use of phytosterols and phytostanols (PS) as food supplements to control plasma cholesterol concentrations has recently received attention as its efficacy has been endorsed by scientific authorities and leading guidelines. However, the effects of phytosterols on lipid profiles and atherosclerosis remain incomplete and controversial. This study aims to investigate the effects of PS supplementation on lipid profiles and apolipoproteins in adults based on a systematic review of the literature and a meta-analysis of randomized controlled trials (RCTs).

**Methods:**

A comprehensive search was conducted for RCTs published in PubMed, Embase, Cochrane Library, and Web of Science as of May 2024. Random effects model was utilized to determine the mean differences and 95% confidence interval for changes in circulating lipid profiles and apolipoproteins.

**Results:**

Twenty-eight RCTs with a total of 1777 participants (895 cases and 882 controls) are included in the qualitative synthesis. PS supplementation significantly reduced total cholesterol (TC), low-density lipoprotein cholesterol (LDL-c), and apolipoprotein B (Apo-B) levels, as well as Apo-B/apolipoprotein A1 ratios, but increased high-density lipoprotein cholesterol levels. PS supplementation dose is associated with TC, LDL-c, and Apo-B levels in a dose–response manner.

**Conclusion:**

Our findings suggest that dietary phytosterols can effectively promote the reduction of TC, LDL-c, and Apo-B, along with increased high-density lipoprotein cholesterol in adults.

## 1. Introduction

The rising incidence of cardiovascular diseases (CVDs) is directly related to the global epidemic of obesity, metabolic syndrome, and diabetes.^[1]^ Dysregulation of blood cholesterol and lipoproteins is recognized as a cardiovascular risk factor for atherosclerotic CVDs.^[2–6]^ Numerous epidemiological or genetic studies and clinical interventions have indisputably demonstrated that low-density lipoproteins (LDL) are causal to the development of atherosclerotic lesions.^[7,8]^ Previous studies have shown that total atherosclerotic plaque burden is proportional to the cumulative exposure to LDL and other Apo-B-containing lipoproteins.^[9]^ Lowering LDL-cholesterol (LDL-c) through dietary changes or applying lipid-lowering agents has been shown to substantially reduce the incidence of CVDs. Furthermore, maintaining LDL-c levels below 40 mg/dL can yield sustained clinical benefits,^[10,11]^ which has been endorsed by the 2019 European Society of Cardiology/European Atherosclerosis Society Dyslipidemia Guidelines.^[12]^ Apo-B, one of the structural lipoproteins located on LDL granules, has shown atherogenic effects. In contrast, apolipoprotein A1 (Apo-A1), the major lipoprotein component of plasma high-density lipoprotein (HDL), is protective against atherosclerosis. Thus, it has been suggested that these apolipoproteins can be used as biomarkers to assess the risk of CVDs.^[13–16]^

Adopting a healthy diet is one of the critical paths to lipid-lowering therapy. Phytosterol and phytostanol are homologs of cholesterol synthesized only by plants and are present in the diet in quantities comparable to cholesterol (200–400 mg per day). Scientific authorities and major guidelines have recognized the ability of phytosterol (PS) to control plasma cholesterol concentrations.^[17–21]^ PS has been shown to reduce LDL-c levels by around 8% to 10% when consumed at a dosage of 2 grams per day (expressed as free plant stanol or plant sterol equivalents).^[1]^ Moreover, a clinical trial demonstrated that orange juice beverages fortified with PS (2 g/day) effectively reduced biomarkers for inflammation, such as serum interleukin-6 and interleukin-1b levels, in healthy subjects and provided lipid profile benefits.^[22]^ The consumption of PS ester spread for 1 month reduced total cholesterol (TC), LDL-c, high sensitivity C-reactive protein, and estimated cardiovascular risk in subjects with mild hypercholesterolemia.^[23]^ These studies suggest that PS may have cardiovascular risk-reducing and anti-inflammatory effects in healthy individuals and patients with hypercholesterolemia. As growing scientific evidence indicates that PS offers multiple cardiovascular protective benefits, many institutions and guidelines have recommended using PS as a dietary supplement. The Food and Drug Administration initially greenlighted health claim endorsements on food labels that contain plant sterol/stanol esters, highlighting their correlation with a diminished risk of coronary heart disease.^[24]^ The recently updated guidelines endorsed by the National Heart Foundation of Australia and European Society of Cardiology/European Atherosclerosis Society have recommended the application of phytosterols as a supplementary strategy alongside lifestyle modifications to decrease blood cholesterol levels.^[12,25]^

Although PS has been found to possess qualities that can lower cholesterol levels and potentially decrease the risk of CVDs, there are apprehensions regarding potential detrimental cardiovascular consequences associated with their consumption as previous clinical trials or case–control studies have reported the associations between elevated serum PS concentrations and increased incidence of CVDs.^[26–29]^ For instance, the PROCAM study revealed that a higher plasma sitosterol concentration is even associated with an increased incidence of coronary events.^[29]^ However, the prospective EPIC-Norfolk Population Study found no significant difference in plasma levels of PS between coronary heart disease patients and healthy controls.^[30]^ This contentious issue is closely linked to the lack of thorough and unified research. Therefore, the German Heart Society remains cautious about endorsing phytosterols as dietary supplements, arguing that emergent evidence indicates the atherogenic properties inherent in phytosterols.^[31]^

Given the relationship between phytosterols and CVDs remains controversial, our study aimed to assess the impact of phytosterol supplements on blood cholesterol levels and lipid profiles by employing a comprehensive and integrated approach, which would assist experts in providing recommendations about using phytosterol supplements for individuals at risk of developing atherosclerosis or CVDs.

## 2. Methods

This systematic review and meta-analysis of randomized controlled trials complied with the Preferred Reporting Items for Systematic Reviews and Meta-Analyses statement.^[32]^

### 2.1. Literature search strategy

An electronic literature search was performed in the PubMed, Embase, Web of Science, and Cochrane Library databases from inception until May 2024 and restricted in English. We searched the databases above using the medical subject headings. Non-mesh terms were searched in combination with search terms relevant to the health outcome of interest: “phytosterol” OR “phytostanol” OR “plant sterol” OR “plant stanol” OR “sitosterol” OR “sitostanol” OR “campesterol” OR “campestanol” OR “stigmasterol” OR “stigmastanol” OR “brassicasterol” combined with “Intervention Studies” OR “intervention” OR “controlled trial” OR “randomized” OR “randomly” OR “placebo” OR “assignment” OR “randomized controlled trial” OR “randomized clinical trial” OR “RCT” OR “blinded” OR “double-blind” OR “double-blinded” OR “trial” OR “controlled clinical trial” OR “pragmatic clinical trial” OR “crossover procedure” OR “crossover trial” OR “double-blind method” OR “equivalence trial” OR “double-blind procedure.” The search was limited to human studies. Keywords were searched in articles’ titles, abstracts, and hedge words. Two authors (Y-F.Z. and W.Q.) searched the electronic databases separately, and disagreements were resolved by group discussion.

### 2.2. Study selection

All articles obtained through electronic or manual search were inputted into the EndNote software (EndNote X6, Thomson Reuters). Initially, duplicate publications were eliminated, followed by a thorough evaluation of each study based on the title and abstract to identify studies that potentially met the eligible criteria. The authors conducted a comprehensive review of the complete text of each paper that successfully passed the initial assessment. All clinical trials assessing the impact of PS supplementation on lipid profiles were ultimately incorporated into the current meta-analysis. Studies were included in the present meta-analysis if they met the following inclusion criteria: (1) conducted in adults; (2) were randomized controlled trials (RCTs) from parallel or cross-over designs; (3) prescribed PS as a supplement or enrichment (studies which supplemented another compound in combination with PS in both intervention and control group also were included); (4) examined the effects of PS on blood and lipid levels; and (5) provided adequate information on lipid levels in the intervention and placebo groups. Studies were excluded if they (1) used a mixture of PS with other substances only in an intervention group; (2) involved taking medications to assist with phytosterol supplementation; (3) were uncontrolled studies or without a placebo; (4) reported duplicate data on the same population; (5) included pregnant or lactating women; (6) were animal studies, conference papers, reviews, letters, editorial articles, or case reports.

### 2.3. Data extraction

The data was collected from eligible articles using a predesigned standardized electronic form. A predesigned excel sheet was used to organize the data into categories by 3 independent authors (Y-F.Z., W.Q., and H.F). The collected information included the last name of the first author, publication year, country of publication, demographic details of participants such as sex and age, study design (including crossover and parallel study), sample size in both control and intervention groups, type, and dosage of intervention in each group, trial duration, and the outcomes investigated which included TC, LDL-c, HDL-cholesterol (HDL-c), Apo-A1, Apo-B, and Apo-B/Apo-A1 ratio.

### 2.4. Quality assessment

Data was entered into the Cochrane Review Manager software (RevMan 5.4.1). The quality of the included studies was evaluated by 2 authors separately, utilizing the Cochrane Collaboration Scale, which included the following items: (1) adequacy of sequence generation (selection bias); (2) configuration hiding (selection bias); (3) blindness (performance deviation); (4) result evaluation blindness (detection bias); (5) clarification failure and incomplete result data (loss deviation); (6) selective reporting results (reporting bias); (7) other possible sources of deviation. As per the Cochrane Handbook,^[33]^ studies are categorized within each domain as having a low risk of bias (L), high risk of bias (H), or unclear risk of bias (U).

### 2.5. Statistical analysis

Circulating concentrations of lipid profiles were collated in millimoles per liter (mmol/L) while circulating concentrations of apolipoproteins were collated in grams per liter (g/L). Mean change and standard deviation at baseline and post-intervention are used in both groups. We calculated the net changes using the differences (intervention minus control) in the changes (final values minus baseline values) in mean values. The effect sizes are expressed as weighted mean difference (WMD) and 95% confidence interval (CI). The random effects model (DerSimonian-Laird methodology) was conducted to calculate the overall effect sizes to minimize the impact of heterogeneity. Cochrane Q and *I*^2^ tests assessed the heterogeneity of the studies. A threshold of *I*^2^ > 40% indicated clinically significant heterogeneity. Potential publication bias was accessed using Begg rank test, Egger test, and funnel plots.^[34]^ Since the magnitude of heterogeneity informs the selection between random-effects and fixed-effects models, we initially used a random-effects model to account for potential variability across studies. Additionally, we performed a leave-one-out sensitivity analysis to assess the robustness of the results under different model assumptions and to evaluate the reliability of the pooled results. Subgroup analyses were performed to investigate potential factors contributing to the variability observed between studies. These factors included phytosterol dosage, intervention duration, and health status. The evaluation of heterogeneity among subgroups was conducted utilizing a fixed-effects model. The impact of phytosterol dosage (grams per day) on potential nonlinear outcomes was evaluated using a restricted cubic spline model. The nonlinear model was successfully fitted, and the resulting outcomes revealed confidence intervals at a 95% significance level. The statistical analyses were conducted using R software (version 4.1.0) with the meta package (version 5.1.1) and the “dosresmeta” package (version 2.0.1).^[35]^ A 2-tailed *P* < .05 was considered statistically significant. This study is a review article and does not require ethical approval.

## 3. Results

### 3.1. Literature search

**Figure 1.**
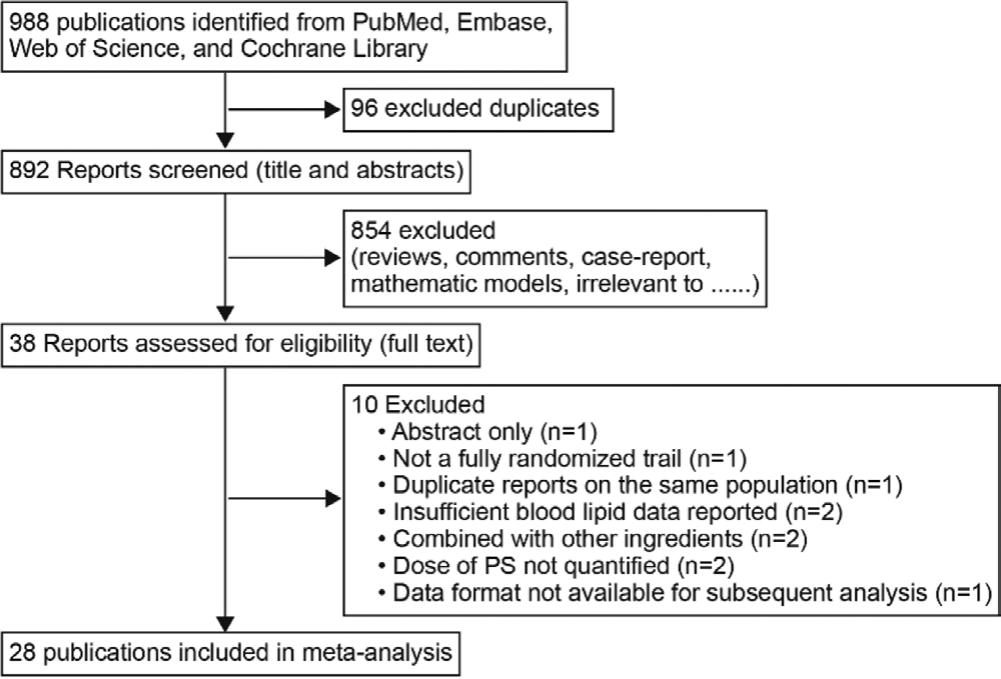
Flow chart of systematic literature search for trials, published through May 2024, that met the study inclusion and exclusion criteria.

The literature search flowchart, following the Preferred Reporting Items for Systematic Review and Meta-Analysis guidelines, is presented in Figure 1. Out of the 988 studies initially selected for the screening process, a subset of 38 full-text articles underwent a comprehensive evaluation. Then, 28 were considered eligible for inclusion in the review.^[36–63]^ Reasons for exclusion included abstract form only (n = 1), not a fully randomized trial (n = 1), duplicate reports on the same population (n = 1), insufficient blood lipid data reported (n = 2), abstract in English but full text in other languages (n = 2), plant stanol/plant sterol were not quantified (n = 2), data were in interval format rather than mean standard deviation format and subsequent analysis was not possible (n = 1).

### 3.2. Characteristics of included studies

Table 1 displays the relevant characteristics of the 28 trials included in the analysis. The reports published between 2000 and 2023 were merged into a total sample size of 1777 participants from trials conducted in Europe (n = 17), Asia (n = 6), North America (n = 4), and South America (n = 1). Participants in 17 studies were in good health and others had prehypertension, hyperlipidemia, familial hypercholesterolemia, and mild or moderate hypercholesterolemia. A total of 17 studies employed a parallel group design, whereas 11 investigations utilized a crossover approach.

**Table 1:** Characteristics of the included studies.

Trial	Country	Design	Dosage	Intervention form	Control	Duration	Age (I, C)	Gender (M/F)	Sample size (I/C)	Health condition
Fuentes, F. (2008)^[36]^	Spain	Crossover	2.5 g/d	Supp.F	Low-fat diets	4-wk	42 ± 18	15/15	60 (30/30)	FH
Buyuktuncer, Z. (2013)^[37]^	Turkey	Parallel	1.9 g/d	Supp.F	Low-fat yoghurt	4-wk	45.5 ± 7.06, 43.5 ± 10.52	15/20 14/21	70 (35/35)	Mild to moderate HCL
Sola, Rosa (2012)^[38]^	Spain	Parallel	2 g/d	Supp.F	Cocoa, hazelnut cream	4-wk	56.03 ± 10.06, 56.79 ± 10.46	13/17 12/16	58 (30/28)	PHTN
Homma, Y. (2003)^[39]^	Japan	Parallel	2 g/d	Supp.F	Low-fat spread	4-wk	47 ± 13, 46 ± 14	11/29 14/21	77 (33/34)	Healthy
Madsen, Martin B. (2007)^[40]^	Denmark	Crossover	2.3 g/d	Supp.F	Spread, milks	4-wk	50.8 ± 9.6, 50.3 ± 10.3	12/36	92 (46/46)	Moderate HCL
Thomsen, A. B. (2004)^[41]^	Denmark	Crossover	1.6 g/d	Supp.F	Milk	4-wk	60 ± 5	18/51	138 (69/69)	HCL
Temme, E. H. (2002)^[42]^	Belgium	Crossover	2.1 g/d	Supp.F	Low-fat spreads	4-wk	55 ± 9	22/20	84 (42/42)	Primary HLD
Castro Cabezas, M. (2006)^[43]^	Netherlands	Parallel	3 g/d	Supp.F	Margarine	6-wk	45.7 ± 11.7, 51.7 ± 15.3	5/6 3/6	20 (11/9)	Primary HLD
de Jong, A. (2008)^[44]^	Netherlands	Parallel	2.5 g/d	Supp.F	Margarine	85-wk	59.2 ± 10.8, 59.9 ± 7.6	10/8 11/6	35 (18/17)	Healthy
Jauhiainen, T. (2006)^[45]^	Finland	Parallel	2 g/d	Supp.F	Cheese	5-wk	43.3 ± 9.5	24/43	67 (33/34)	HCL
Hernandez-Mijares, Antonio (2010)^[46]^	Spain	Parallel	2 g/d	Supp.F	Low-fat milk	3-m	50 ± 8, 49 ± 10	9/22 6/18	55 (31/24)	Mild HCL
De Jong, A. (2008)^[47]^	Netherlands	Parallel	2.5 g/d	Supp.F	Margarine	16-wk	58.4 ± 9, 57.8 ± 5.8	8/7 4/7	26 (15/11)	Moderate HCL
Banuls, Celia (2010)^[48]^	Spain	Parallel	2 g/d	Supp.F	Low-fat milk	3-m	50.0 ± 10.2	NR	40 (20/20)	Healthy
Colgan, H. A. (2004)^[49]^	Ireland	Parallel	1.6 g/d	Supp.F	Reduced fat margarine	3-wk	44.1 ± 7.6, 48.5 ± 9.8	27/21	96 (48/48)	HCL
Khandelwal, S. (2009)^[50]^	India	Parallel	2 g/d	Supp.F	Yoghurt	4-wk	45.9 ± 0.9, 46.1 ± 0.9	41/6 41/5	93 (47/46)	Healthy
Ottestad, I. (2013)^[51]^	Norway	Crossover	2 g/d	Capsules	Sunflower oil	4-wk	58 ± 9, 62 ± 9	12/9 12/8	41 (21/20)	Healthy
Chen, S. C. (2009)^[52]^	USA	Crossover	3.3 g/d	Supp.F	Salad dressings, spreads	4-wk	51.7 ± 2.4	13/9	44 (22/22)	Healthy
Ruiu, G. (2009)^[53]^	Italy	Crossover	1 g/d	Supp.F	Fermented pasteurized milk	4-wk	54.2 ± 7.3	10/5	30 (15/15)	Healthy
Takeshita, Masao (2008)^[54]^	Japan	Parallel	0.5 g/d	Supp.F	TAG oil	12-wk	61 ± 8, 58 ± 6	3/11 3/12	29 (14/15)	Healthy
Sialvera, T. E. (2012)^[55]^	Greece	Parallel	4 g/d	Supp.F	Yogurt	2-m	30–65	29/24 31/24	108 (53/55)	MetS
Theuwissen, E. (2009)^[56]^	Netherlands	Parallel	2.5 g/d	Supp.F	Margarine	3-wk	54 ± 8	16/12	28 (14/14)	HTG
Plat, J. (2009)^[57]^	Netherlands	Parallel	2 g/d	Supp.F	Yogurt	9-wk	60 ± 4, 60 ± 7	5/4 7/2	18 (9/9)	HF
Judd, J. T. (2002)^[58]^	USA	Crossover	2.2 g/d	Supp.F	Salad dressing	3-wk	47.1 ± 1.54	26/27	106 (53/53)	Normal to mild HCL
Nunes, V. S. (2022)^[59]^	Brazil	Crossover	1.6 g/d	Supp.F	Soy milk	4-wk	58 ± 12	7/31	76 (38/38)	Healthy
Devaraj, S. (2006)^[60]^	USA	Parallel	2 g/d	Supp.F	Orange juice	8-wk	44 ± 14, 48 ± 15	16/20 15/21	72 (36/36)	Healthy
Theuwissen, E. (2007)^[61]^	Netherlands	Crossover	1.5 g/d	Supp.F	β-Glucan muesli	4-wk	18–65	NR	86 (43/43)	Healthy
Seki, Shinji (2003)^[62]^	Japan	Parallel	0.45 g/d	Supp.F	Oil	4-wk	41 ± 9.9, 43.5 ± 1.9	NR	44 (22/22)	Healthy
Shaghaghi, Mandana Amir (2014)^[63]^	Canada	Crossover	2 g/d	Supp.F	Yogurt	4-wk	50 ± 2.1	25/22	94 (47/47)	Mild to moderate HTG

C = control, g/d = gram per day, HCL = hypercholesterolemia, HF = familial hypercholesterolemia, HLD = hyperlipidemia, HTG = hypertriglyceridemia, I = intervention, m = month, MetS = metabolic syndrome, NR = not reported, PHTN = prehypertension, PS = phytosterol, Supp.F = supplemental food, TAG = triacylglycerol, wk = week.

### 3.3. Quality assessment

**Figure 2.**
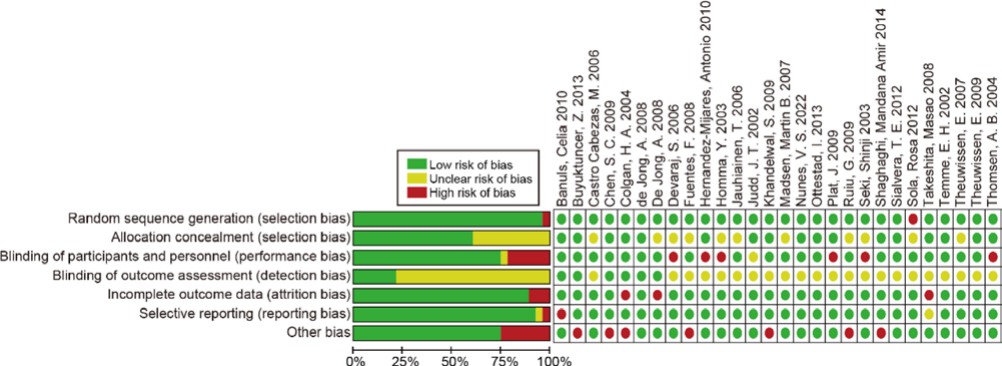
The quality assessment of included studies. Review authors’ judgements about each risk of bias item are presented as percentages across all included studies.

The results of the quality assessment for the eligible studies are delineated in Figure 2. Most parameters employed in risk assessment indicated either a low or ambiguous bias risk within the incorporated trials. Six of the 28 studies included in this analysis adhered to double-blinding protocols, while 15 were evaluated as single-blinded. This evaluation was based on the absence of information or insufficient information regarding the blinding of outcome assessment.

### 3.4. Results of meta-analysis

#### 3.4.1. The effects of PS supplementation on total cholesterol

**Figure 3.**
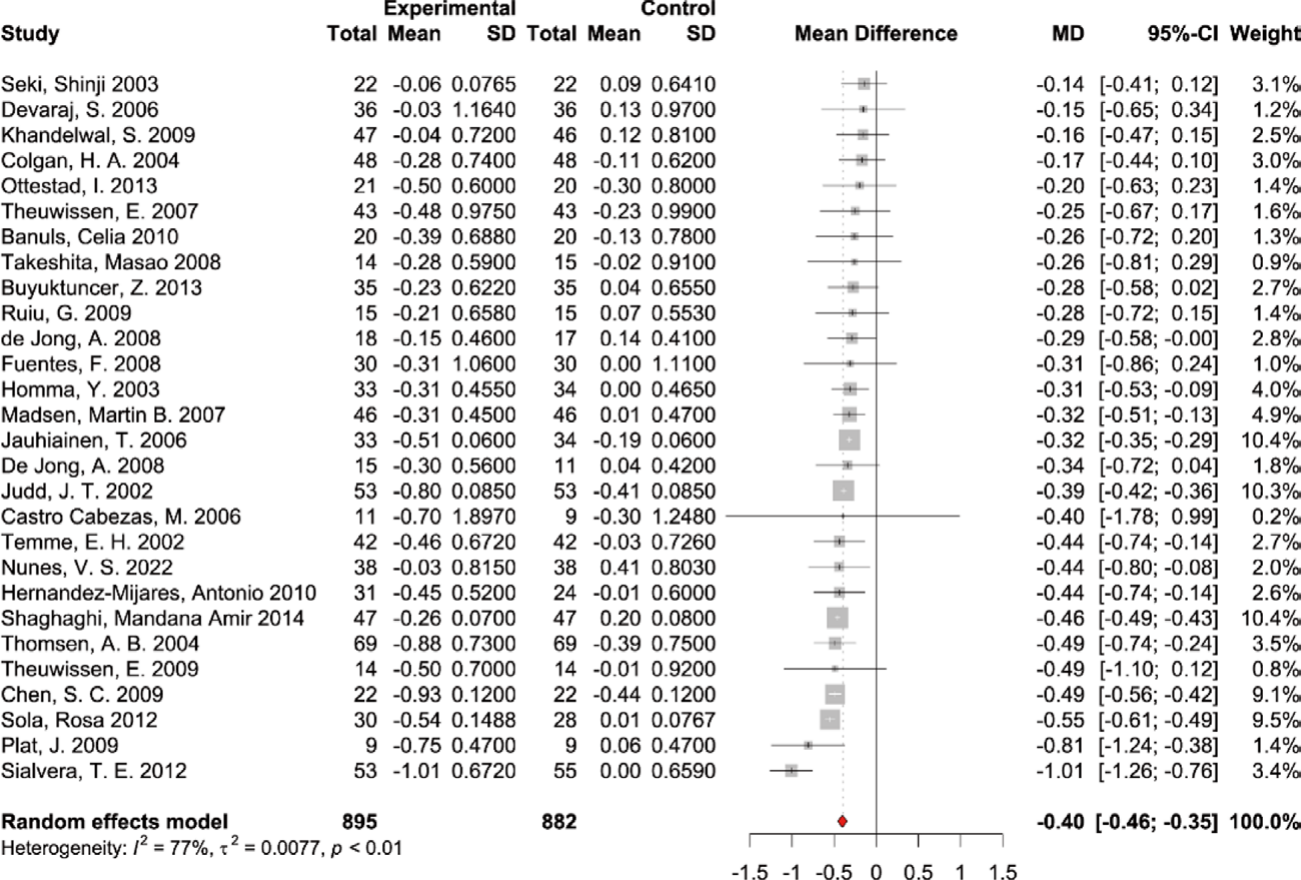
Forest plot of randomized controlled trials investigating the effects of PS supplementation on total cholesterol.

**Figure 4.**
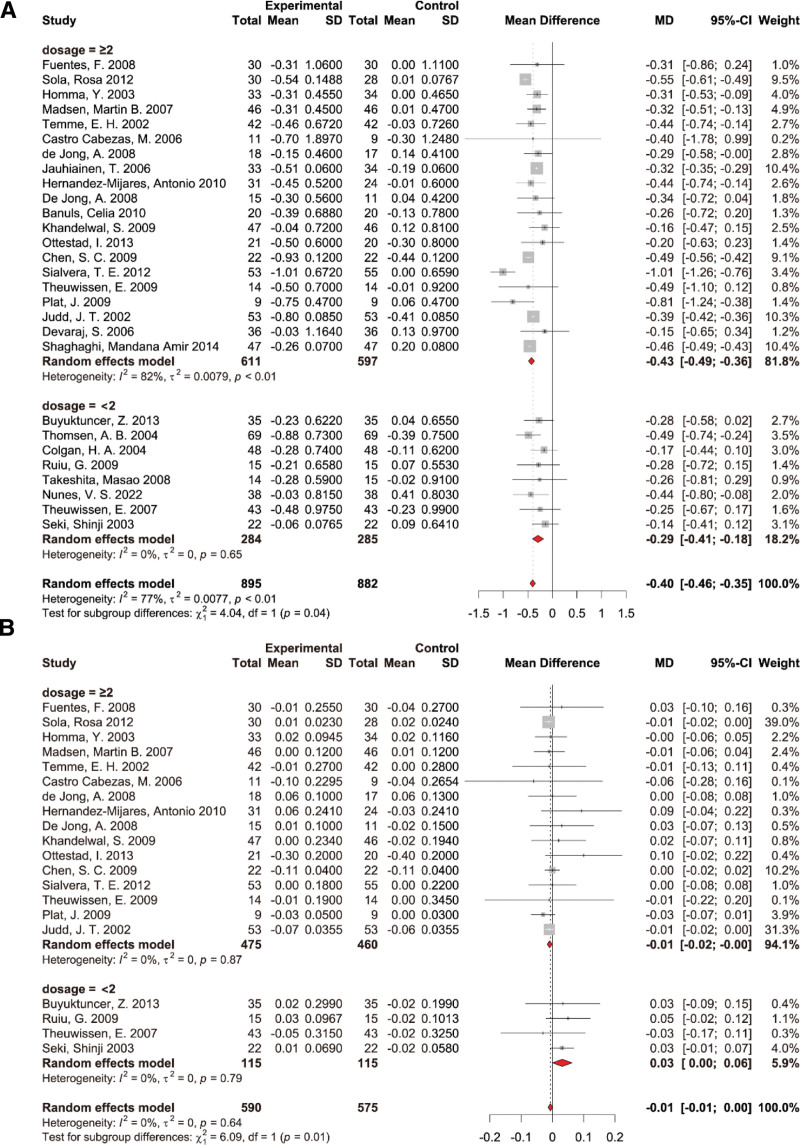
Results of subgroup analysis of dosage on (A) total cholesterol and (B) apolipoprotein-A1.

A total of 28 studies, encompassing 1777 participants (895 cases and 882 controls), provided data on TC measures. A random-effects model was used to calculate pooled effects due to the significant heterogeneity between studies. The results of the pooled analysis demonstrated a substantial decrease in TC levels with the supplementation of PS (WMD: −0.40 mmol/L, 95% CI: [‐0.46, −0.34], *P* < .01), with significant heterogeneity among studies (*I*^2^ = 77%, *P* < .01) (Fig. 3). Removing individual studies did not materially change the pooled estimation of PS supplementation effect on TC (see Fig. S1, Supplemental Digital Content, http://links.lww.com/MD/N703, leave-one-out sensitivity analysis for the effect of PS supplementation on plasma total cholesterol). Subgroup analyses were performed based on PS dosage (<2 g/d and ≥2 g/d), intervention duration (<4 w and ≥4 w) (see Fig. S10, Supplemental Digital Content, http://links.lww.com/MD/N703, results of subgroup analysis of duration on TC), and health status (see Fig. S7, Supplemental Digital Content, http://links.lww.com/MD/N703, results of subgroup analysis of health condition on TC) of the participants. Subgroup analysis showed a significant difference between PS supplemental dosage subgroups (*P* for subgroup difference = .04); the PS intervention effect on TC was −0.43 mmol/L (95% CI: [‐0.49, −0.36]) in the high-dosage subgroup (≥2 g/d) and −0.29 mmol/L (95% CI: [‐0.41, −0.18]) in the low-dosage subgroup (<2 g/d) (Fig. 4A). The results indicated intervention time and health status of the participants did not have a statistically significant impact on the effectiveness of PS supplementation in reducing TC levels.

#### 3.4.2. The effects of PS supplementation on LDL-c

**Figure 5.**
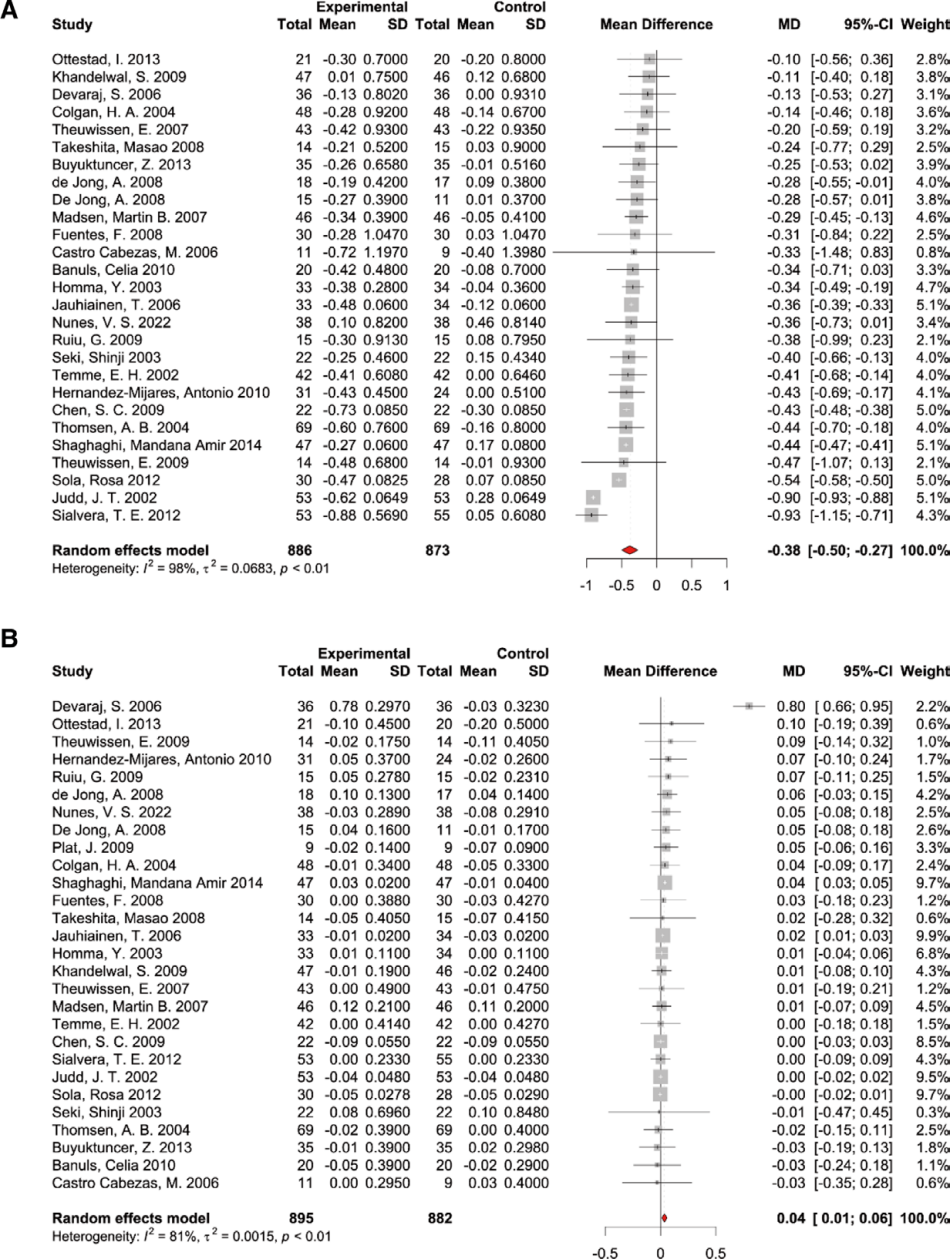
Forest plot of randomized controlled trials investigating the effects of PS supplementation on (A) low-density lipoprotein cholesterol and (B) high-density lipoprotein cholesterol.

From the 27 studies analyzed, which included 1759 participants (886 cases and 873 controls), data on LDL-c measures was obtained. Given the notable heterogeneity across studies, we employed a random-effects model for pooled effect calculation. This analysis showed a marked reduction in LDL-c levels upon PS supplementation (WMD: −0.38 mmol/L, 95% CI: [‐0.50, −0.27], *P* < .01), with significant heterogeneity observed (*I*^2^ =98%, *P* < .01) (Fig. 5A). Removing individual studies did not materially change the pooled estimation of PS supplementation effect on LDL-c (see Fig. S2A, Supplemental Digital Content, http://links.lww.com/MD/N703, leave-one-out sensitivity analysis for the effect of PS supplementation on LDL-c). Subgroup analyses were performed based on PS dosage (<2 g/d and ≥2 g/d) (see Fig. S4, Supplemental Digital Content, http://links.lww.com/MD/N703, results of subgroup analysis of dosage on LDL-c), intervention duration (<4 w and ≥4 w) (see Fig. S11, Supplemental Digital Content, http://links.lww.com/MD/N703, results of subgroup analysis of duration on LDL-c), and health condition (see Fig. S8, Supplemental Digital Content, http://links.lww.com/MD/N703, results of subgroup analysis of health condition on LDL-c) of the study population. The results indicated that the participants’ dosage, duration, and health condition did not have a statistically significant impact on the effectiveness of PS supplementation in reducing LDL-c levels.

#### 3.4.3. The effects of PS supplementation on HDL-c

**Figure 6.**
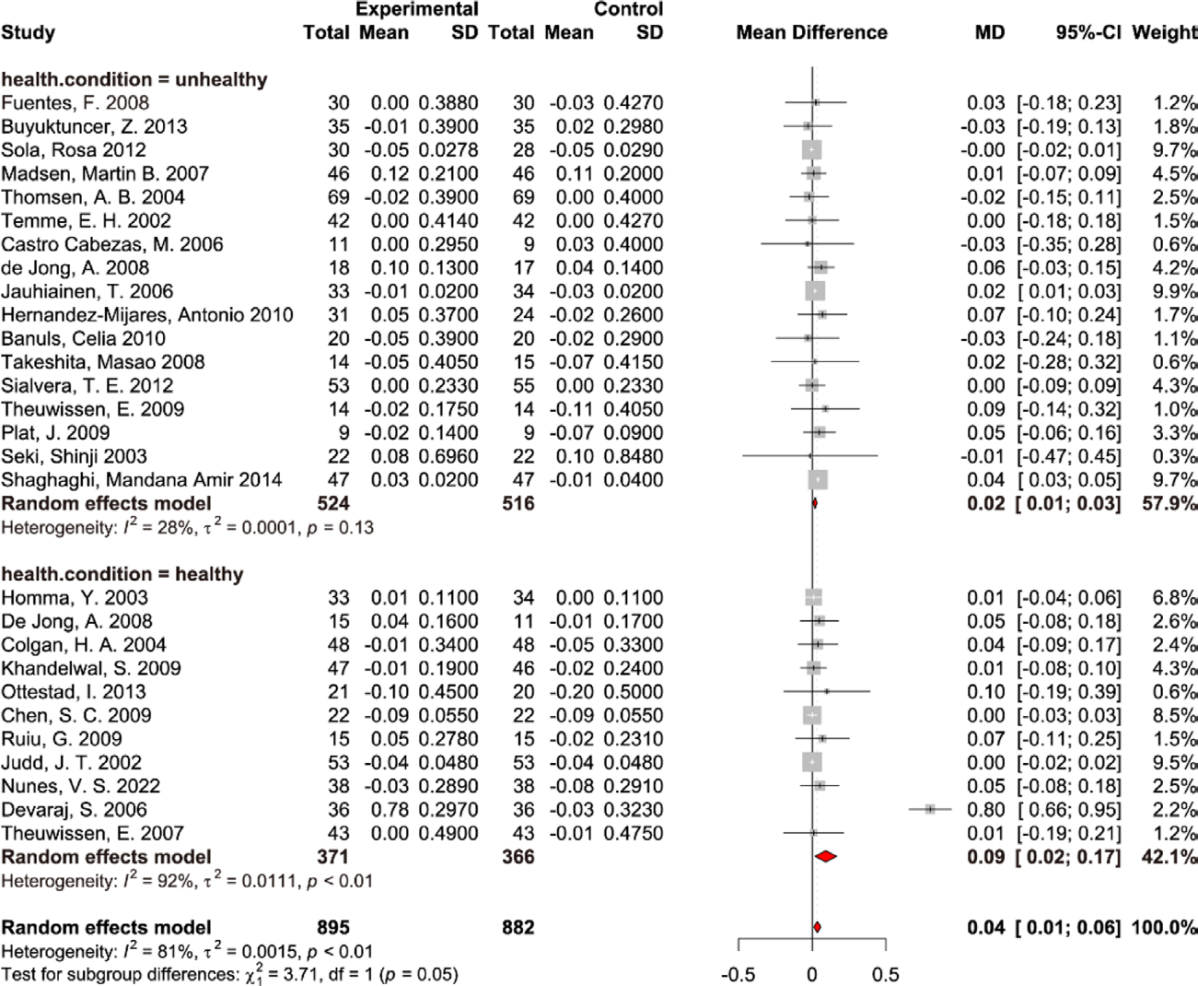
Results of subgroup analysis of health condition on high-density lipoprotein cholesterol.

A total of 28 studies, encompassing 1777 participants (895 cases and 882 controls), provided data on HDL-c measures. Because of the significant heterogeneity between studies, a random-effects model was used to calculate pooled effects. The results of the pooled analysis demonstrated a substantial increase in HDL-c levels with the supplementation of PS (WMD: 0.04 mmol/L, 95% CI: [0.01, 0.06], *P* < .01) with significant heterogeneity among studies (*I*^2^ = 81%, *P* < .01) (Fig. 5B). Removing individual studies did not materially change the pooled estimation of PS supplementation effect on HDL-c (see Fig. S2B, Supplemental Digital Content, http://links.lww.com/MD/N703, leave-one-out sensitivity analysis for the effect of PS supplementation on HDL-c.). Subgroup analyses were performed based on PS dosage (<2 g/d and ≥2 g/d) (see Fig. S5, Supplemental Digital Content, http://links.lww.com/MD/N703, results of subgroup analysis of dosage on HDL-c), intervention duration (<4 w and ≥4 w) (see Fig. S12, Supplemental Digital Content, http://links.lww.com/MD/N703, results of subgroup analysis of dosage on HDL-c), and the health condition of the participants. Further, subgroup examinations were segmented by PS dosage, intervention duration, and the health condition of the participants. Notably, there was a significant distinction based on participants’ health condition (*P* for subgroup difference = .05) (Fig. 6). The impact of PS on HDL-c was 0.02 mmol/L (95% CI: [0.01, 0.03]) for the unhealthy subgroup and 0.09 mmol/L (95% CI: [0.02, 0.17]) for the healthy subgroup. The dosage and duration of the intervention were found not to exert a statistically significant influence on the efficacy of PS in enhancing HDL-c levels.

#### 3.4.4. The effects of PS supplementation on Apo-A1

**Figure 7.**
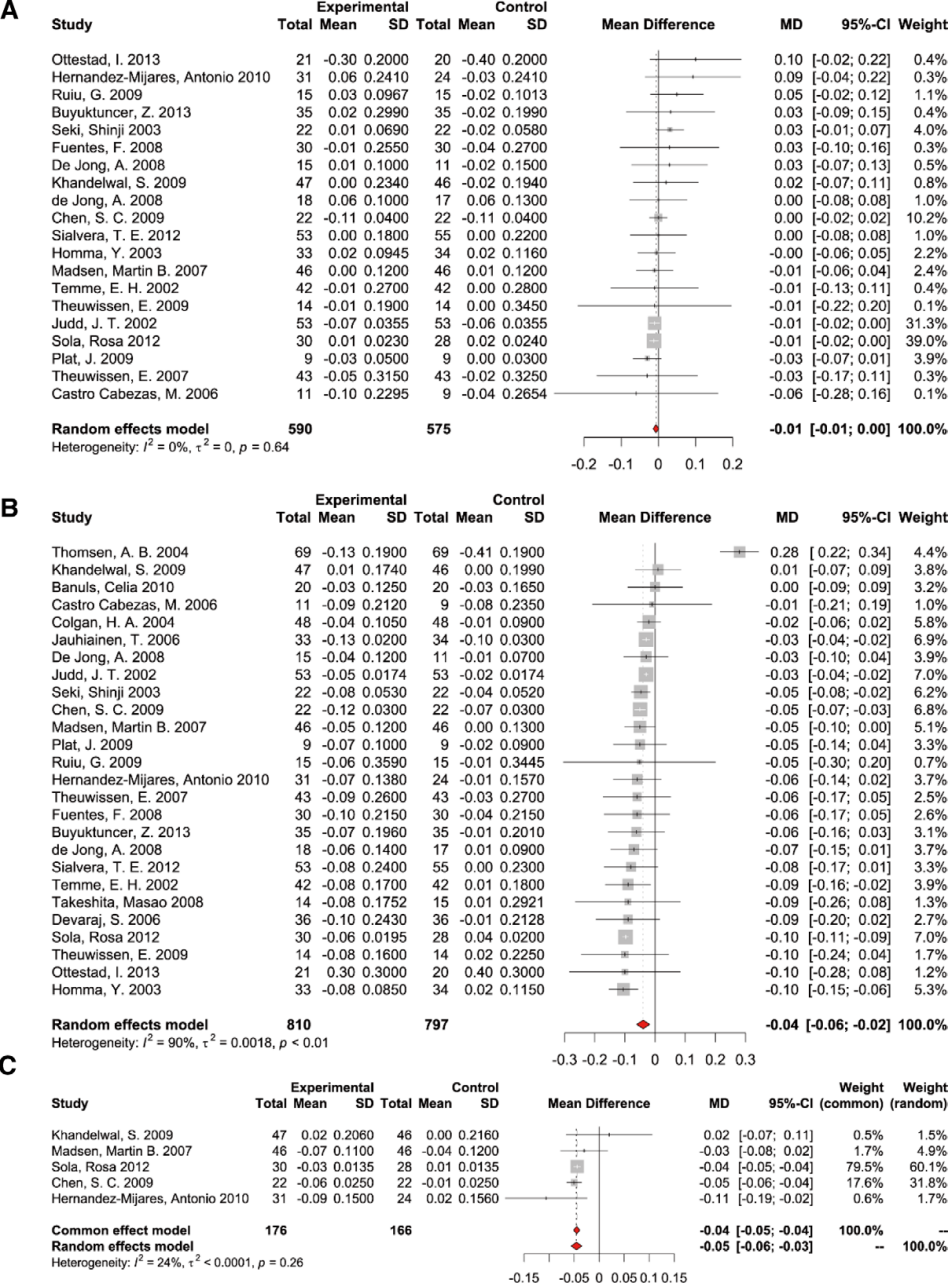
Forest plot of randomized controlled trials investigating the effects of PS supplementation on (A) apolipoprotein-A1, (B) apolipoprotein-B, and (C) apolipoprotein-B/apolipoprotein-A1 ratio.

Data was obtained from a collection of 20 studies for 1165 participants, divided into 590 cases and 575 controls, focusing on Apo-A1 measures. The pooled analysis revealed a marked decline in Apo-A1 levels when supplemented with PS (WMD: −0.01 g/L, 95% CI: [‐0.01,0], *P* = .64), but without notable heterogeneity across the studies (*I*^2^ = 0, *P* > .05) (Fig. 7A). Removing individual studies did not materially change the pooled estimation of PS supplementation effect on Apo-A1 (see Fig. S3A, Supplemental Digital Content, http://links.lww.com/MD/N703, leave-one-out sensitivity analysis for the effect of PS supplementation on Apo-A1). Subgroup analyses were performed based on PS dosage (<2 g/d and ≥2 g/d), intervention duration (<4 w and ≥4 w) (see Fig. S13A, Supplemental Digital Content, http://links.lww.com/MD/N703, results of subgroup analysis of duration on Apo-A1), and health condition (see Fig. S9A, Supplemental Digital Content, http://links.lww.com/MD/N703, results of subgroup analysis of health condition on Apo-A1) of the study population. Subgroup analysis showed a significant difference between PS supplemental dosage subgroups (*P* for subgroup difference = .01). The PS intervention effect on Apo-A1 was −0.01 mmol/L (95% CI: [‐0.02, 0]) in the high-dosage subgroup (≥2 g/d) and 0.03 mmol/L (95% CI: [0, 0.06]) in the low-dosage subgroup (<2 g/d) (Fig. 4B). The duration of the intervention and the health condition of the participants were found not to exert a statistically significant influence on the efficacy of PS in increasing Apo-A1 levels.

#### 3.4.5. The effects of PS supplementation on Apo-B

A total of 26 studies, encompassing 1607 participants (810 cases and 797 controls), provided data on apolipoprotein-B (Apo-B) measures. Because of the significant heterogeneity between studies, a random-effects model was used to calculate pooled effects. The results of the pooled analysis demonstrated a substantial decrease in Apo-B levels with the supplementation of PS (WMD: −0.04 g/L, 95% CI: [‐0.06, −0.02], *P* < .01) with significant heterogeneity among studies (*I*^2^ = 90%, *P* < .01) (Fig. 7B). Removing individual studies did not materially change the pooled estimation of PS supplementation effect on Apo-B (see Fig. S3B, Supplemental Digital Content, http://links.lww.com/MD/N703, leave-one-out sensitivity analysis for the effect of PS supplementation on Apo-B). Detailed subgroup assessments, categorized based on PS dosage (<2 g/d and ≥2 g/d) (see Fig. S6, Supplemental Digital Content, http://links.lww.com/MD/N703, results of subgroup analysis of dosage on Apo-B), intervention duration (<4 w and ≥4 w) (see Fig. S13B, Supplemental Digital Content, http://links.lww.com/MD/N703, results of subgroup analysis of duration on Apo-B), and participants’ health status (see Fig. S9B, Supplemental Digital Content, http://links.lww.com/MD/N703, results of subgroup analysis of duration on Apo-B), revealed that neither the dosage, duration of intervention nor the health condition of participants significantly influenced the efficacy of PS in lowering Apo-B levels.

#### 3.4.6. The effects of PS supplementation on Apo-B/Apo-A1 ratio

A total of 5 studies, encompassing 342 participants (176 cases and 166 controls), provided data on Apo-B/Apo-A1 ratio measures. The results of the pooled analysis demonstrated a substantial decrease in Apo-B/Apo-A1 ratio levels with the supplementation of PS (WMD: −0.04, 95% CI: [‐0.05, −0.04], *P* < .01) with nonsignificant heterogeneity among studies (*I*^2^ = 24%, *P* = .26) (Fig. 7C). Subgroup analyses were not conducted due to the small sample size.

#### 3.4.7. Dose–response relationships between PS supplementation and lipid profiles

**Figure 8.**
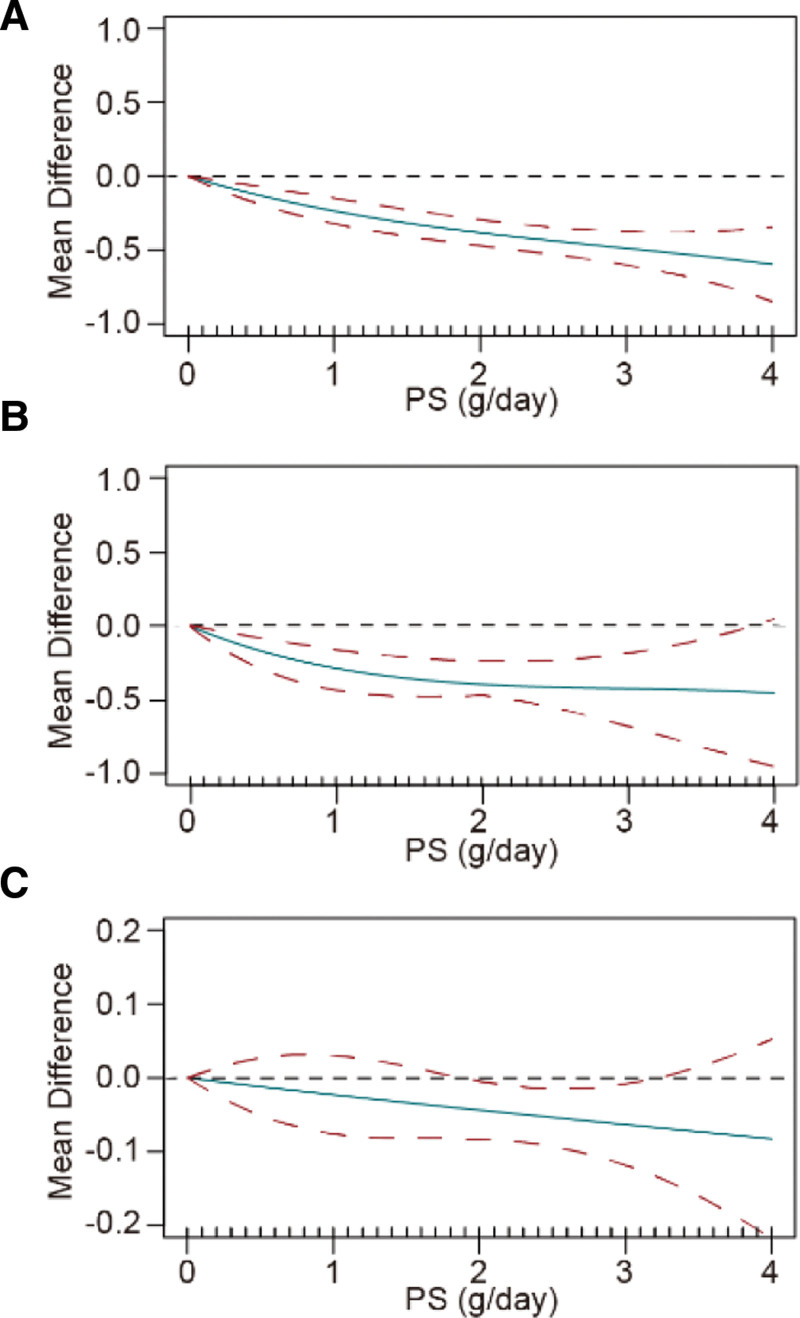
Nonlinear dose-response relationship between PS supplementation and mean differences in (A) total cholesterol, (B) low-density lipoprotein, and (C) apolipoprotein-B. The dotted lines represent 95% confidence intervals.

The dose–response analysis indicated a significant association between PS supplementation and TC (*χ*^2^ = 238.17, *P* < .001), LDL-c (*χ*^2^ = 93.99, *P* < .001), and Apo-B (*χ*^2^ = 16.89, *P* < .001) (Fig. 8). However, the levels of HDL-c (*χ*^2^ = 3.297, *P* = .192) and Apo-A1 (*χ*^2^ = 3.702, *P* = .157) did not exhibit a nonlinear relationship with the dosage of PS (g/d).

### 3.5. Publication bias

**Figure 9.**
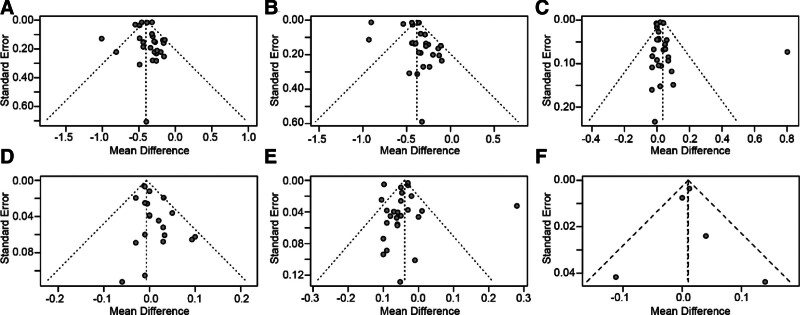
Funnel plot revealing publication bias in the studies reporting the effects of PS supplementation on plasma lipid profiles including (A) total cholesterol, (B) low-density lipoprotein cholesterol, (C) high-density lipoprotein cholesterol, (D) apolipoprotein-A1, (E) apolipoprotein-B, and (F) apolipoprotein-B/apolipoprotein-A1 ratio.

The evaluation of publication bias in the meta-analysis of PS consumption on TC, LDL-c, HDL-c, Apo-A1, Apo-B, and Apo-B/Apo-A1 ratio was conducted using visual inspection of the funnel plot (Fig. 9), Egger regression asymmetry tests, and Begg test. The results of these assessments indicated no evidence of publication bias in TC, HDL-c, and Apo-B (*P* = .9370 for TC, *P* = .1983 for HDL-c, and *P* = .8781 for Apo-B). However, Egger regression asymmetry tests showed a remarkable presence of publication bias for Apo-A1 (*t* = 2.46, *f* = 18, *P* = .0245), although Begg test did not reveal any substantial evidence of publication bias (z = 0.65, *P* = .5164). In addition, the result of Begg test demonstrated a significant publication bias for LDL-c (z = ‐2.11, *P* = .0352), but Egger regression asymmetry tests did not yield statistically significant evidence of publication bias (*t* = 1.32, df = 25, *P* = .1983). These results suggest that publication bias is a possible cause of heterogeneity among study results.

## 4. Discussion

In this study, we conducted a systematic review and meta-analysis of randomized controlled clinical trials to explore the influence of PS supplementation on lipid profiles and apolipoproteins in the whole population. Our comprehensive assessment demonstrated that TC, LDL-c, and Apo-B levels and Apo-B/Apo-A1 ratios decreased significantly following the administration of PS as supplements. Conversely, HDL-c levels exhibited a substantial increase. Furthermore, the dose-response analyses highlight negative correlations between PS supplementation and TC, LDL-c, and Apo-B levels. However, the levels of HDL-c and Apo-A1 did not exhibit a nonlinear relationship with the dosage of PS (g/d). Consequently, our results underscore the potential clinical benefits of PS consumption in mitigating the risk of atherosclerosis and the associated CVDs.

Dyslipidemia, defined as an abnormal level of lipids in the blood, is well-recognized as being associated with a multitude of diseases, including coronary artery disease, stroke, diabetes, and nonalcoholic fatty liver disease.^[18,64,65]^ It is well-established that elevated TC and LDL-c levels are positively associated with the incidence and mortality of CVDs, while HDL-c level is negatively correlated.^[66,67]^ Apolipoproteins are also strongly associated with atherosclerosis and other CVDs.^[68–70]^ Numerous cohort studies and clinical research have demonstrated a significant correlation between decreased LDL-c levels and a lower risk of developing CVDs, including atherosclerosis and coronary heart disease.^[20,71–73]^ Lowering LDL-c has been proven to be one of the pivotal strategies for mitigating the risk of CVDs.^[74,75]^ Furthermore, given that HDL-c primarily reverses cholesterol transport, an elevation in HDL-c levels is typically associated with a reduced risk of CVDs. Therefore, using PS as a food supplement could be a rational approach to control blood cholesterol concentrations and reduce the risk of atherosclerosis. A previous clinical trial demonstrated that incorporating phytosterols into ezetimibe enhanced its impact on whole-body cholesterol metabolism and circulating LDL cholesterol levels, underscoring the potential benefits of dietary phytosterols as a supplementary treatment against CVDs and dyslipidemia.^[76]^

Previous meta-analyses have primarily focused on the effect of PS on TC and LDL-c, with limited attention given to apolipoproteins.^[77,78]^ Apo-A1, a major component of HDL-c, is crucial for cholesterol efflux from tissues to the liver, a process known as reverse cholesterol transport.^[70,79–81]^ Although no discernible correlation between PS supplements and Apo-A1 was observed, our findings demonstrated a substantial increase in HDL-c levels. This effect may be attributed to PS’s specific influence on cholesterol metabolism rather than directly impacting Apo-A1 expression or turnover.^[82]^ Apo-B is present in all non-HDL cholesterol particles, including LDL-c and very low-density lipoprotein cholesterol. Higher Apo-B levels indicate increased atherogenic particles, elevating the risk of atherosclerosis and CVDs.^[70,79]^ Moreover, the Apo-B/Apo-A1 ratio has been extensively explored as a potential cardiovascular risk predictor.^[83]^ Therefore, our meta-analyses reveal the potential implications of PS supplementation on lipid profiles and apolipoproteins and provide promising insights for early CVDs prevention.

The study population comprised both healthy individuals and individuals with hypercholesterolemia, hypertension, and metabolic syndrome. Our findings indicate that PS supplementation substantially reduced total TC, LDL-c, and ApoB while concurrently enhancing HDL-c across the entire population, encompassing both healthy individuals and those with hypercholesterolemia, hypertension, and metabolic syndrome. Furthermore, subgroup analysis based on health conditions unveiled a relatively more substantial effect of PS supplementation on HDL-c enhancement in healthy individuals as compared to patients; in contrast, there were no significant differences observed in the effect of PS on other lipid profiles and apolipoproteins among the various health condition subgroups. Prior meta-analyses, which primarily concentrated on specific patient populations such as hypercholesterolemia, familial hypercholesterolemia, or postmenopausal women, have consistently reported substantial reductions in LDL-c and TC levels but no noteworthy effects on HDL-C.^[77,78,84,85]^ These findings underscore the preventive significance of PS consumption in the context of CVDs.

In addition, the dosage and duration of PS supplementation are critical in achieving favorable outcomes over time. Our study conducted a dose–response meta-analysis to elucidate the potential optimal dosage of PS supplementation that improves lipid profiles and apolipoproteins, contributing a valuable reference for forthcoming RCTs that aim to investigate the dose–response relationship between PS supplementation and lipid profiles as well as apolipoproteins. Our subgroup analysis revealed that daily consumption of >2 g PS confers a statistically significant benefit to TC and HDL-c levels, reinforcing the existing recommendations for PS consumption. A previous dose–response meta-analysis indicated a plateau in the LDL-c reduction at an approximate PS intake of 3 g/d.^[86]^ Consistent with this, our study findings also suggest that the impact of PS intake on TC and LDL-c begins to plateau once the dosage reaches 3 g/d. Nevertheless, it is crucial to recognize that the impact of higher doses of PS supplementation (≥4 g/d) on lipid profiles remains unclear. Moreover, among the 28 clinical trials in our study, the intervention durations ranged from 3 to 85-wk, with only 5 extending beyond 3 months. The long-term effects of PS supplementation warrant further investigation. Furthermore, although these studies measured lipid profiles and other metabolites, the absence of investigations exploring the association between PS intake and clinical endpoints such as cardiovascular events and mortality hampers a comprehensive assessment of the preventive effects of PS on CVDs. Additional clinical trials are imperative to assess the effects and potential risks associated with higher doses and longer durations of PS intake.

Various potential mechanisms have been postulated to elucidate the potential benefits of PS supplementation on the modulation of circulating lipids and cholesterol. Phytosterols are a class of lipid-soluble triterpenes with similar structural properties to cholesterol. Phytosterols, either consumed from dietary sources or as supplements, undergo absorption by the human intestine after their incorporation into mixed micelles. Micelles facilitate the transportation of phytosterols into enterocytes via the Niemann-Pick C1-Like 1 pathway.^[87]^ The absorption of cholesterol from the gastrointestinal tract occurs via the same route utilized by phytosterols, resulting in a competitive interaction between phytosterols and cholesterol for absorption within enterocytes. Hence, the absorption of cholesterol fractions declines as the quantity of phytosterols in the gastrointestinal tract surges.^[88]^ Moreover, phytosterols can restrict cholesterol uptake by directly forming co-crystals with cholesterol within the intestinal lumen and promoting its elimination through the fecal route. Apart from its known role in cholesterol absorption regulation, the precise impact of PS on other cell types, such as endothelial cells, cardiomyocytes, and immune cells, remains unclear.

Several limitations of the present study warrant consideration. Most included studies featured limited sample sizes, and a noticeable degree of heterogeneity was present. This heterogeneity implies that the effect size may differ among studies due to variations in study designs, study populations, interventions, or outcomes. Furthermore, given that PS constitutes a diverse mixture rather than a singular compound, the evaluation of the effects of specific classes of PS poses a significant challenge. Therefore, further research is essential to elucidate the distinct functions associated with various categories of PS. Lastly, another noteworthy limitation is the lack of clinical trials involving high doses and prolonged durations of PS interventions.

## 5. Conclusion

In summary, this extensive systematic review and meta-analysis have provided evidence indicating that PS supplementation may lead to a reduction in TC, LDL-c, and Apo-B levels while simultaneously boosting HDL-c levels. However, the presence of significant heterogeneity across the studies suggested the need for additional RCTs to elucidate the association between PS supplementation and lipid profiles and CVDs.

## Acknowledgments

The authors thank Mr. Zhihao Liu and Prof Yan Yang for their valuable advice on the project.

## Author contributions

**Conceptualization:** Jinzhao Yang, Yang Zhang.

**Data curation:** Yi-Feng Zhang, Hanxiao Feng, Kuan Jiang.

**Formal analysis:** Yi-Feng Zhang, Wanning Qiao, Hanxiao Feng, Kuan Jiang.

**Funding acquisition:** Yang Zhang.

**Investigation:** Yi-Feng Zhang.

**Methodology:** Yi-Feng Zhang, Wanning Qiao, Hanxiao Feng, Kuan Jiang, Tao Zhou, Yang Zhang.

**Project administration:** Yang Zhang.

**Resources:** Yi-Feng Zhang, Wanning Qiao, Yang Zhang.

**Software:** Yi-Feng Zhang.

**Supervision:** Tao Zhou, Yang Zhang.

**Validation:** Tao Zhou, Yang Zhang.

**Visualization:** Yang Zhang.

**Writing – original draft:** Yi-Feng Zhang, Yang Zhang.

**Writing – review & editing:** Yi-Feng Zhang, Wanning Qiao, Jinzhao Yang, Yang Zhang.

## Supplementary Material


