# Supplementary material for: Effects of phytosterol supplementation on lipid profiles and apolipoproteins: A meta-analysis of randomized controlled trials
Source: Medicine (Baltimore). 2024 Oct 18;103(42):e40020. doi: 10.1097/MD.0000000000040020 (PMC11495771; doi:10.1097/MD.0000000000040020)

Supplemental Figure 1

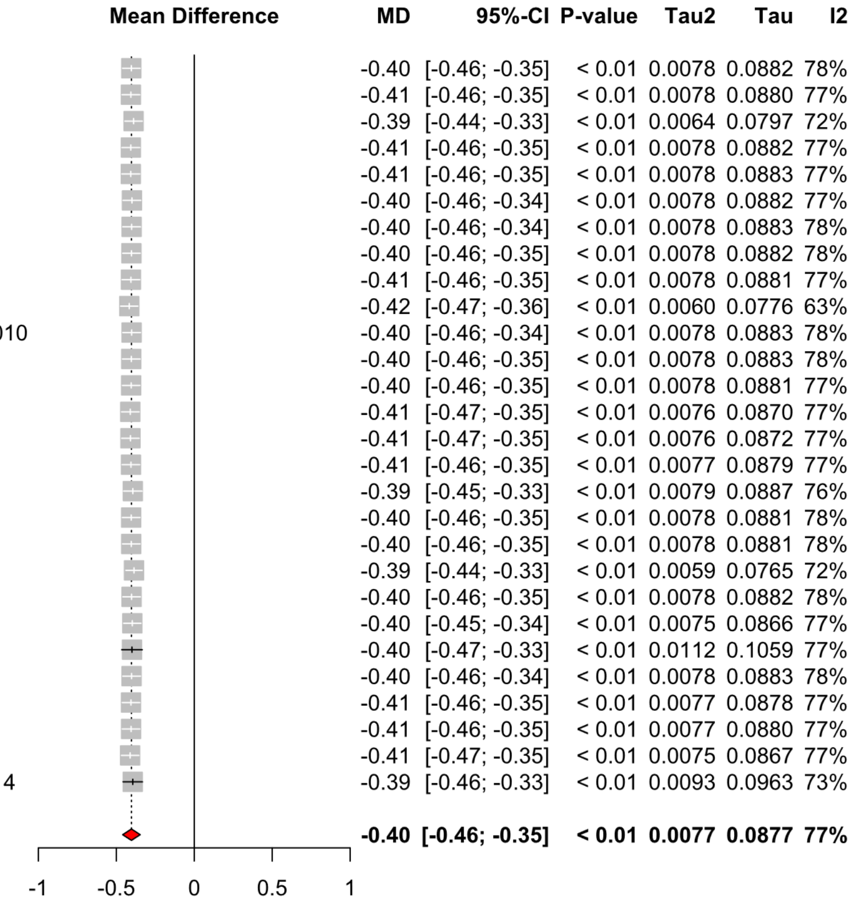

Study	MD	95%-CI	P-value	Tau2	Tau	I2
Omitting Fuentes, F. 2008	-0.40	[-0.46; -0.35]	< 0.01	0.0078	0.0882	78%
Omitting Buyuktuncer, Z. 2013	-0.41	[-0.46; -0.35]	< 0.01	0.0078	0.0880	77%
Omitting Sola, Rosa 2012	-0.39	[-0.44; -0.33]	< 0.01	0.0064	0.0797	72%
Omitting Homma, Y. 2003	-0.41	[-0.46; -0.35]	< 0.01	0.0078	0.0882	77%
Omitting Madsen, Martin B. 2007	-0.41	[-0.46; -0.35]	< 0.01	0.0078	0.0883	77%
Omitting Thomsen, A. B. 2004	-0.40	[-0.46; -0.34]	< 0.01	0.0078	0.0882	77%
Omitting Temme, E. H. 2002	-0.40	[-0.46; -0.34]	< 0.01	0.0078	0.0883	78%
Omitting Castro Cabezas, M. 2006	-0.40	[-0.46; -0.35]	< 0.01	0.0078	0.0882	78%
Omitting de Jong, A. 2008	-0.41	[-0.46; -0.35]	< 0.01	0.0078	0.0881	77%
Omitting Jauhiainen, T. 2006	-0.42	[-0.47; -0.36]	< 0.01	0.0060	0.0776	63%
Omitting Hernandez-Mijares, Antonio 2010	-0.40	[-0.46; -0.34]	< 0.01	0.0078	0.0883	78%
Omitting De Jong, A. 2008	-0.40	[-0.46; -0.35]	< 0.01	0.0078	0.0883	78%
Omitting Banuls, Celia 2010	-0.40	[-0.46; -0.35]	< 0.01	0.0078	0.0881	77%
Omitting Colgan, H. A. 2004	-0.41	[-0.47; -0.35]	< 0.01	0.0076	0.0870	77%
Omitting Khandelwal, S. 2009	-0.41	[-0.47; -0.35]	< 0.01	0.0076	0.0872	77%
Omitting Ottestad, I. 2013	-0.41	[-0.46; -0.35]	< 0.01	0.0077	0.0879	77%
Omitting Chen, S. C. 2009	-0.39	[-0.45; -0.33]	< 0.01	0.0079	0.0887	76%
Omitting Ruiu, G. 2009	-0.40	[-0.46; -0.35]	< 0.01	0.0078	0.0881	78%
Omitting Takeshita, Masao 2008	-0.40	[-0.46; -0.35]	< 0.01	0.0078	0.0881	78%
Omitting Sialvera, T. E. 2012	-0.39	[-0.44; -0.33]	< 0.01	0.0059	0.0765	72%
Omitting Theuwissen, E. 2009	-0.40	[-0.46; -0.35]	< 0.01	0.0078	0.0882	78%
Omitting Plat, J. 2009	-0.40	[-0.45; -0.34]	< 0.01	0.0075	0.0866	77%
Omitting Judd, J. T. 2002	-0.40	[-0.47; -0.33]	< 0.01	0.0112	0.1059	77%
Omitting Nunes, V. S. 2022	-0.40	[-0.46; -0.34]	< 0.01	0.0078	0.0883	78%
Omitting Devaraj, S. 2006	-0.41	[-0.46; -0.35]	< 0.01	0.0077	0.0878	77%
Omitting Theuwissen, E. 2007	-0.41	[-0.46; -0.35]	< 0.01	0.0077	0.0880	77%
Omitting Seki, Shinji 2003	-0.41	[-0.47; -0.35]	< 0.01	0.0075	0.0867	77%
Omitting Shaghaghi, Mandana Amir 2014	-0.39	[-0.46; -0.33]	< 0.01	0.0093	0.0963	73%
**Random effects model**	**-0.40**	**[-0.46; -0.35]**	**< 0.01**	**0.0077**	**0.0877**	**77%**

Supplemental Figure 2

A

Study	MD	95%-CI	P-value	Tau2	Tau	I2
Omitting Fuentes, F. 2008	-0.39	[-0.50; -0.27]	< 0.01	0.0684	0.2615	98%
Omitting Buyuktuncer, Z. 2013	-0.39	[-0.51; -0.27]	< 0.01	0.0683	0.2614	98%
Omitting Sola, Rosa 2012	-0.37	[-0.50; -0.25]	< 0.01	0.0801	0.2829	98%
Omitting Homma, Y. 2003	-0.39	[-0.50; -0.27]	< 0.01	0.0687	0.2621	98%
Omitting Madsen, Martin B. 2007	-0.39	[-0.51; -0.27]	< 0.01	0.0684	0.2616	98%
Omitting Thomsen, A. B. 2004	-0.38	[-0.50; -0.26]	< 0.01	0.0686	0.2620	98%
Omitting Temme, E. H. 2002	-0.38	[-0.50; -0.26]	< 0.01	0.0686	0.2619	98%
Omitting Castro Cabezas, M. 2006	-0.38	[-0.50; -0.27]	< 0.01	0.0684	0.2615	98%
Omitting de Jong, A. 2008	-0.39	[-0.51; -0.27]	< 0.01	0.0684	0.2615	98%
Omitting Jauhiainen, T. 2006	-0.38	[-0.51; -0.26]	< 0.01	0.0723	0.2689	97%
Omitting Hernandez-Mijares, Antonio 2010	-0.38	[-0.50; -0.26]	< 0.01	0.0686	0.2619	98%
Omitting De Jong, A. 2008	-0.39	[-0.51; -0.27]	< 0.01	0.0684	0.2615	98%
Omitting Banuls, Celia 2010	-0.39	[-0.50; -0.27]	< 0.01	0.0684	0.2616	98%
Omitting Colgan, H. A. 2004	-0.39	[-0.51; -0.28]	< 0.01	0.0681	0.2610	98%
Omitting Khandelwal, S. 2009	-0.40	[-0.51; -0.28]	< 0.01	0.0680	0.2608	98%
Omitting Ottestad, I. 2013	-0.39	[-0.51; -0.28]	< 0.01	0.0682	0.2612	98%
Omitting Chen, S. C. 2009	-0.38	[-0.50; -0.26]	< 0.01	0.0746	0.2732	98%
Omitting Ruiu, G. 2009	-0.38	[-0.50; -0.27]	< 0.01	0.0684	0.2616	98%
Omitting Takeshita, Masao 2008	-0.39	[-0.50; -0.27]	< 0.01	0.0684	0.2615	98%
Omitting Sialvera, T. E. 2012	-0.36	[-0.48; -0.24]	< 0.01	0.0682	0.2611	98%
Omitting Theuwissen, E. 2009	-0.38	[-0.50; -0.27]	< 0.01	0.0684	0.2616	98%
Omitting Judd, J. T. 2002	-0.39	[-0.45; -0.34]	< 0.01	0.0061	0.0783	72%
Omitting Nunes, V. S. 2022	-0.38	[-0.50; -0.27]	< 0.01	0.0685	0.2616	98%
Omitting Devaraj, S. 2006	-0.39	[-0.51; -0.28]	< 0.01	0.0682	0.2612	98%
Omitting Theuwissen, E. 2007	-0.39	[-0.51; -0.27]	< 0.01	0.0683	0.2613	98%
Omitting Seki, Shinji 2003	-0.38	[-0.50; -0.27]	< 0.01	0.0686	0.2619	98%
Omitting Shaghaghi, Mandana Amir 2014	-0.38	[-0.51; -0.25]	< 0.01	0.0872	0.2953	97%
**Random effects model**	**-0.38**	**[-0.50; -0.27]**	**< 0.01**	**0.0683**	**0.2614**	**98%**

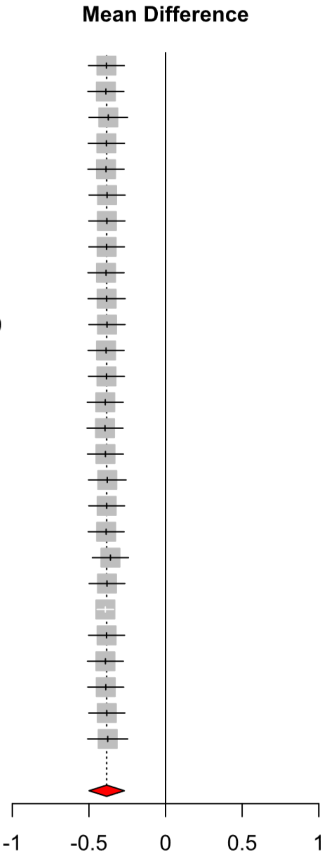

B

Study	MD	95%-CI	P-value	Tau2	Tau	I2
Omitting Fuentes, F. 2008	0.04	[0.01; 0.06]	< 0.01	0.0015	0.0390	82%
Omitting Buyuktuncer, Z. 2013	0.04	[0.01; 0.06]	< 0.01	0.0015	0.0390	82%
Omitting Sola, Rosa 2012	0.04	[0.01; 0.07]	< 0.01	0.0018	0.0430	80%
Omitting Homma, Y. 2003	0.04	[0.01; 0.06]	< 0.01	0.0016	0.0394	82%
Omitting Madsen, Martin B. 2007	0.04	[0.01; 0.06]	< 0.01	0.0015	0.0391	82%
Omitting Thomsen, A. B. 2004	0.04	[0.01; 0.06]	< 0.01	0.0015	0.0390	82%
Omitting Temme, E. H. 2002	0.04	[0.01; 0.06]	< 0.01	0.0015	0.0390	82%
Omitting Castro Cabezas, M. 2006	0.04	[0.01; 0.06]	< 0.01	0.0015	0.0390	82%
Omitting de Jong, A. 2008	0.03	[0.01; 0.06]	< 0.01	0.0015	0.0390	82%
Omitting Jauhiainen, T. 2006	0.04	[0.01; 0.07]	< 0.01	0.0026	0.0506	82%
Omitting Hernandez-Mijares, Antonio 2010	0.03	[0.01; 0.06]	< 0.01	0.0015	0.0390	82%
Omitting De Jong, A. 2008	0.03	[0.01; 0.06]	< 0.01	0.0015	0.0390	82%
Omitting Banuls, Celia 2010	0.04	[0.01; 0.06]	< 0.01	0.0015	0.0390	82%
Omitting Colgan, H. A. 2004	0.04	[0.01; 0.06]	< 0.01	0.0015	0.0390	82%
Omitting Khandelwal, S. 2009	0.04	[0.01; 0.06]	< 0.01	0.0015	0.0391	82%
Omitting Ottestad, I. 2013	0.03	[0.01; 0.06]	< 0.01	0.0015	0.0389	82%
Omitting Chen, S. C. 2009	0.04	[0.01; 0.06]	< 0.01	0.0016	0.0399	82%
Omitting Ruiu, G. 2009	0.03	[0.01; 0.06]	< 0.01	0.0015	0.0390	82%
Omitting Takeshita, Masao 2008	0.04	[0.01; 0.06]	< 0.01	0.0015	0.0390	82%
Omitting Sialvera, T. E. 2012	0.04	[0.01; 0.06]	< 0.01	0.0015	0.0391	82%
Omitting Theuwissen, E. 2009	0.03	[0.01; 0.06]	< 0.01	0.0015	0.0389	82%
Omitting Plat, J. 2009	0.03	[0.01; 0.06]	< 0.01	0.0015	0.0390	82%
Omitting Judd, J. T. 2002	0.04	[0.01; 0.07]	< 0.01	0.0018	0.0420	81%
Omitting Nunes, V. S. 2022	0.03	[0.01; 0.06]	< 0.01	0.0015	0.0390	82%
Omitting Devaraj, S. 2006	0.02	[0.01; 0.02]	< 0.01	< 0.0001	0.0063	11%
Omitting Theuwissen, E. 2007	0.04	[0.01; 0.06]	< 0.01	0.0015	0.0390	82%
Omitting Seki, Shinji 2003	0.04	[0.01; 0.06]	< 0.01	0.0015	0.0390	82%
Omitting Shaghaghi, Mandana Amir 2014	0.04	[0.01; 0.06]	< 0.01	0.0020	0.0443	80%
**Random effects model**	**0.04**	**[0.01; 0.06]**	**< 0.01**	**0.0015**	**0.0388**	**81%**

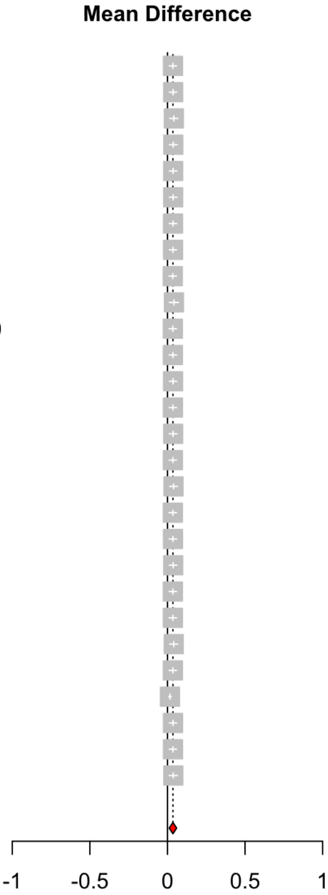

Supplemental Figure 3

A

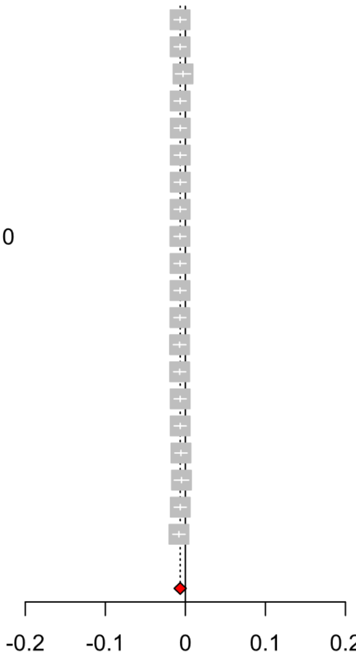

Study	MD	95%-CI	P-value	Tau2	Tau	I2
Omitting Fuentes, F. 2008	-0.01	[-0.01; 0.00]	0.09	0	0	0%
Omitting Buyuktuncer, Z. 2013	-0.01	[-0.01; 0.00]	0.09	0	0	0%
Omitting Sola, Rosa 2012	-0.00	[-0.01; 0.01]	0.55	0	0	0%
Omitting Homma, Y. 2003	-0.01	[-0.01; 0.00]	0.09	0	0	0%
Omitting Madsen, Martin B. 2007	-0.01	[-0.01; 0.00]	0.10	0	0	0%
Omitting Temme, E. H. 2002	-0.01	[-0.01; 0.00]	0.09	0	0	0%
Omitting Castro Cabezas, M. 2006	-0.01	[-0.01; 0.00]	0.10	0	0	0%
Omitting de Jong, A. 2008	-0.01	[-0.01; 0.00]	0.09	0	0	0%
Omitting Hernandez-Mijares, Antonio 2010	-0.01	[-0.01; 0.00]	0.08	0	0	0%
Omitting De Jong, A. 2008	-0.01	[-0.01; 0.00]	0.08	0	0	0%
Omitting Khandelwal, S. 2009	-0.01	[-0.01; 0.00]	0.08	0	0	0%
Omitting Ottestad, I. 2013	-0.01	[-0.01; 0.00]	0.07	0	0	0%
Omitting Chen, S. C. 2009	-0.01	[-0.02; 0.00]	0.08	0	0	0%
Omitting Ruiu, G. 2009	-0.01	[-0.01; 0.00]	0.07	0	0	0%
Omitting Sialvera, T. E. 2012	-0.01	[-0.01; 0.00]	0.09	0	0	0%
Omitting Theuwissen, E. 2009	-0.01	[-0.01; 0.00]	0.09	0	0	0%
Omitting Plat, J. 2009	-0.01	[-0.01; 0.00]	0.16	0	0	0%
Omitting Judd, J. T. 2002	-0.00	[-0.01; 0.00]	0.29	0	0	0%
Omitting Theuwissen, E. 2007	-0.01	[-0.01; 0.00]	0.10	0	0	0%
Omitting Seki, Shinji 2003	-0.01	[-0.02; -0.00]	0.04	0	0	0%
**Random effects model**	**-0.01**	**[-0.01; 0.00]**	**0.09**	**0**	**0**	**0%**

Mean Difference

B

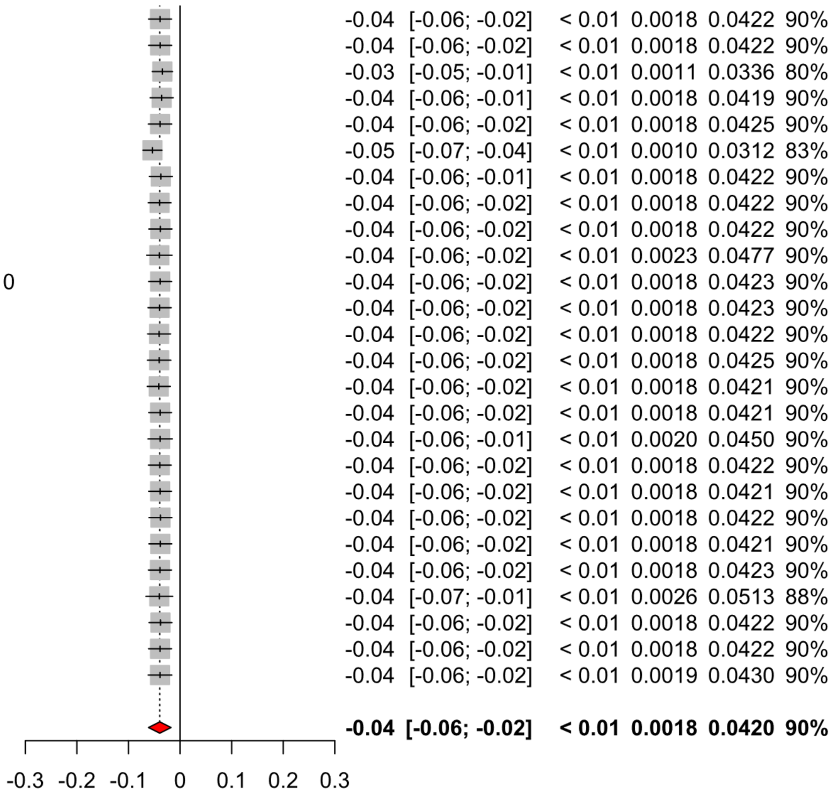

Study	MD	95%-CI	P-value	Tau2	Tau	I2
Omitting Fuentes, F. 2008	-0.04	[-0.06; -0.02]	< 0.01	0.0018	0.0422	90%
Omitting Buyuktuncer, Z. 2013	-0.04	[-0.06; -0.02]	< 0.01	0.0018	0.0422	90%
Omitting Sola, Rosa 2012	-0.03	[-0.05; -0.01]	< 0.01	0.0011	0.0336	80%
Omitting Homma, Y. 2003	-0.04	[-0.06; -0.01]	< 0.01	0.0018	0.0419	90%
Omitting Madsen, Martin B. 2007	-0.04	[-0.06; -0.02]	< 0.01	0.0018	0.0425	90%
Omitting Thomsen, A. B. 2004	-0.05	[-0.07; -0.04]	< 0.01	0.0010	0.0312	83%
Omitting Temme, E. H. 2002	-0.04	[-0.06; -0.01]	< 0.01	0.0018	0.0422	90%
Omitting Castro Cabezas, M. 2006	-0.04	[-0.06; -0.02]	< 0.01	0.0018	0.0422	90%
Omitting de Jong, A. 2008	-0.04	[-0.06; -0.02]	< 0.01	0.0018	0.0422	90%
Omitting Jauhiainen, T. 2006	-0.04	[-0.06; -0.02]	< 0.01	0.0023	0.0477	90%
Omitting Hernandez-Mijares, Antonio 2010	-0.04	[-0.06; -0.02]	< 0.01	0.0018	0.0423	90%
Omitting De Jong, A. 2008	-0.04	[-0.06; -0.02]	< 0.01	0.0018	0.0423	90%
Omitting Banuls, Celia 2010	-0.04	[-0.06; -0.02]	< 0.01	0.0018	0.0422	90%
Omitting Colgan, H. A. 2004	-0.04	[-0.06; -0.02]	< 0.01	0.0018	0.0425	90%
Omitting Khandelwal, S. 2009	-0.04	[-0.06; -0.02]	< 0.01	0.0018	0.0421	90%
Omitting Ottestad, I. 2013	-0.04	[-0.06; -0.02]	< 0.01	0.0018	0.0421	90%
Omitting Chen, S. C. 2009	-0.04	[-0.06; -0.01]	< 0.01	0.0020	0.0450	90%
Omitting Ruiu, G. 2009	-0.04	[-0.06; -0.02]	< 0.01	0.0018	0.0422	90%
Omitting Takeshita, Masao 2008	-0.04	[-0.06; -0.02]	< 0.01	0.0018	0.0421	90%
Omitting Sialvera, T. E. 2012	-0.04	[-0.06; -0.02]	< 0.01	0.0018	0.0422	90%
Omitting Theuwissen, E. 2009	-0.04	[-0.06; -0.02]	< 0.01	0.0018	0.0421	90%
Omitting Plat, J. 2009	-0.04	[-0.06; -0.02]	< 0.01	0.0018	0.0423	90%
Omitting Judd, J. T. 2002	-0.04	[-0.07; -0.01]	< 0.01	0.0026	0.0513	88%
Omitting Devaraj, S. 2006	-0.04	[-0.06; -0.02]	< 0.01	0.0018	0.0422	90%
Omitting Theuwissen, E. 2007	-0.04	[-0.06; -0.02]	< 0.01	0.0018	0.0422	90%
Omitting Seki, Shinji 2003	-0.04	[-0.06; -0.02]	< 0.01	0.0019	0.0430	90%
**Random effects model**	**-0.04**	**[-0.06; -0.02]**	**< 0.01**	**0.0018**	**0.0420**	**90%**

Mean Difference

Supplemental Figure 4

Study	Experimental Total	Experimental Mean	Experimental SD	Control Total	Control Mean	Control SD	MD	95%-CI	Weight
**dosage = ≥2**									
Fuentes, F. 2008	30	-0.28	1.0470	30	0.03	1.0470	-0.31	[-0.84; 0.22]	2.5%
Sola, Rosa 2012	30	-0.47	0.0825	28	0.07	0.0850	-0.54	[-0.58; -0.50]	5.0%
Homma, Y. 2003	33	-0.38	0.2800	34	-0.04	0.3600	-0.34	[-0.49; -0.19]	4.7%
Madsen, Martin B. 2007	46	-0.34	0.3900	46	-0.05	0.4100	-0.29	[-0.45; -0.13]	4.6%
Temme, E. H. 2002	42	-0.41	0.6080	42	0.00	0.6460	-0.41	[-0.68; -0.14]	4.0%
Castro Cabezas, M. 2006	11	-0.72	1.1970	9	-0.40	1.3980	-0.33	[-1.48; 0.83]	0.8%
de Jong, A. 2008	18	-0.19	0.4200	17	0.09	0.3800	-0.28	[-0.55; -0.01]	4.0%
Jauhiainen, T. 2006	33	-0.48	0.0600	34	-0.12	0.0600	-0.36	[-0.39; -0.33]	5.1%
Hernandez-Mijares, Antonio 2010	31	-0.43	0.4500	24	0.00	0.5100	-0.43	[-0.69; -0.17]	4.1%
De Jong, A. 2008	15	-0.27	0.3900	11	0.01	0.3700	-0.28	[-0.57; 0.01]	3.8%
Banuls, Celia 2010	20	-0.42	0.4800	20	-0.08	0.7000	-0.34	[-0.71; 0.03]	3.3%
Khandelwal, S. 2009	47	0.01	0.7500	46	0.12	0.6800	-0.11	[-0.40; 0.18]	3.8%
Ottestad, I. 2013	21	-0.30	0.7000	20	-0.20	0.8000	-0.10	[-0.56; 0.36]	2.8%
Chen, S. C. 2009	22	-0.73	0.0850	22	-0.30	0.0850	-0.43	[-0.48; -0.38]	5.0%
Sialvera, T. E. 2012	53	-0.88	0.5690	55	0.05	0.6080	-0.93	[-1.15; -0.71]	4.3%
Theuwissen, E. 2009	14	-0.48	0.6800	14	-0.01	0.9300	-0.47	[-1.07; 0.13]	2.1%
Judd, J. T. 2002	53	-0.62	0.0649	53	0.28	0.0649	-0.90	[-0.93; -0.88]	5.1%
Devaraj, S. 2006	36	-0.13	0.8020	36	0.00	0.9310	-0.13	[-0.53; 0.27]	3.1%
Shaghaghi, Mandana Amir 2014	47	-0.27	0.0600	47	0.17	0.0800	-0.44	[-0.47; -0.41]	5.1%
**Random effects model**	**602**			**588**			**-0.41**	**[-0.55; -0.28]**	**73.3%**
Heterogeneity: $I^2 = 98\%$, $\tau^2 = 0.0689$, $p < 0.01$									
**dosage = <2**									
Buyuktuncer, Z. 2013	35	-0.26	0.6580	35	-0.01	0.5160	-0.25	[-0.53; 0.02]	3.9%
Thomsen, A. B. 2004	69	-0.60	0.7600	69	-0.16	0.8000	-0.44	[-0.70; -0.18]	4.0%
Colgan, H. A. 2004	48	-0.28	0.9200	48	-0.14	0.6700	-0.14	[-0.46; 0.18]	3.6%
Ruiu, G. 2009	15	-0.30	0.9130	15	0.08	0.7950	-0.38	[-0.99; 0.23]	2.1%
Takeshita, Masao 2008	14	-0.21	0.5200	15	0.03	0.9000	-0.24	[-0.77; 0.29]	2.5%
Nunes, V. S. 2022	38	0.10	0.8200	38	0.46	0.8140	-0.36	[-0.73; 0.01]	3.4%
Theuwissen, E. 2007	43	-0.42	0.9300	43	-0.22	0.9350	-0.20	[-0.59; 0.19]	3.2%
Seki, Shinji 2003	22	-0.25	0.4600	22	0.15	0.4340	-0.40	[-0.66; -0.13]	4.0%
**Random effects model**	**284**			**285**			**-0.32**	**[-0.43; -0.20]**	**26.7%**
Heterogeneity: $I^2 = 0\%$, $\tau^2 = 0$, $p = 0.88$									
**Random effects model**	**886**			**873**			**-0.38**	**[-0.50; -0.27]**	**100.0%**
Heterogeneity: $I^2 = 98\%$, $\tau^2 = 0.0683$, $p < 0.01$									
Test for subgroup differences: $\chi^2_1 = 1.13$, df = 1 ($p = 0.29$)									

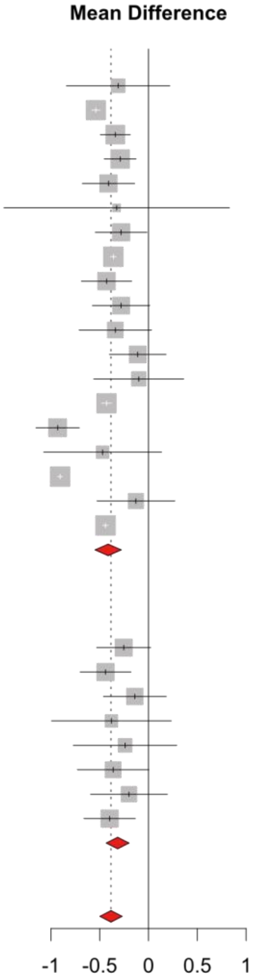

Supplemental Figure 5

Study	Experimental Total	Experimental Mean	Experimental SD	Control Total	Control Mean	Control SD	MD	95%-CI	Weight
**dosage = ≥2**									
Fuentes, F. 2008	30	0.00	0.3880	30	-0.03	0.4270	0.03	[-0.18; 0.23]	1.2%
Sola, Rosa 2012	30	-0.05	0.0278	28	-0.05	0.0290	-0.00	[-0.02; 0.01]	9.7%
Homma, Y. 2003	33	0.01	0.1100	34	0.00	0.1100	0.01	[-0.04; 0.06]	6.8%
Madsen, Martin B. 2007	46	0.12	0.2100	46	0.11	0.2000	0.01	[-0.07; 0.09]	4.5%
Temme, E. H. 2002	42	0.00	0.4140	42	0.00	0.4270	0.00	[-0.18; 0.18]	1.5%
Castro Cabezas, M. 2006	11	0.00	0.2950	9	0.03	0.4000	-0.03	[-0.35; 0.28]	0.6%
de Jong, A. 2008	18	0.10	0.1300	17	0.04	0.1400	0.06	[-0.03; 0.15]	4.2%
Jauhiainen, T. 2006	33	-0.01	0.0200	34	-0.03	0.0200	0.02	[0.01; 0.03]	9.9%
Hernandez-Mijares, Antonio 2010	31	0.05	0.3700	24	-0.02	0.2600	0.07	[-0.10; 0.24]	1.7%
De Jong, A. 2008	15	0.04	0.1600	11	-0.01	0.1700	0.05	[-0.08; 0.18]	2.6%
Banuls, Celia 2010	20	-0.05	0.3900	20	-0.02	0.2900	-0.03	[-0.24; 0.18]	1.1%
Khandelwal, S. 2009	47	-0.01	0.1900	46	-0.02	0.2400	0.01	[-0.08; 0.10]	4.3%
Ottestad, I. 2013	21	-0.10	0.4500	20	-0.20	0.5000	0.10	[-0.19; 0.39]	0.6%
Chen, S. C. 2009	22	-0.09	0.0550	22	-0.09	0.0550	0.00	[-0.03; 0.03]	8.5%
Sialvera, T. E. 2012	53	0.00	0.2330	55	0.00	0.2330	0.00	[-0.09; 0.09]	4.3%
Theuwissen, E. 2009	14	-0.02	0.1750	14	-0.11	0.4050	0.09	[-0.14; 0.32]	1.0%
Plat, J. 2009	9	-0.02	0.1400	9	-0.07	0.0900	0.05	[-0.06; 0.16]	3.3%
Judd, J. T. 2002	53	-0.04	0.0480	53	-0.04	0.0480	0.00	[-0.02; 0.02]	9.5%
Devaraj, S. 2006	36	0.78	0.2970	36	-0.03	0.3230	0.80	[0.66; 0.95]	2.2%
Shaghaghi, Mandana Amir 2014	47	0.03	0.0200	47	-0.01	0.0400	0.04	[0.03; 0.05]	9.7%
**Random effects model**	**611**			**597**			**0.04**	**[0.01; 0.06]**	**87.1%**
Heterogeneity: I^2 = 87%, τ^2 = 0.0016, p < 0.01									
**dosage = <2**									
Buyuktuncer, Z. 2013	35	-0.01	0.3900	35	0.02	0.2980	-0.03	[-0.19; 0.13]	1.8%
Thomsen, A. B. 2004	69	-0.02	0.3900	69	0.00	0.4000	-0.02	[-0.15; 0.11]	2.5%
Colgan, H. A. 2004	48	-0.01	0.3400	48	-0.05	0.3300	0.04	[-0.09; 0.17]	2.4%
Ruiu, G. 2009	15	0.05	0.2780	15	-0.02	0.2310	0.07	[-0.11; 0.25]	1.5%
Takeshita, Masao 2008	14	-0.05	0.4050	15	-0.07	0.4150	0.02	[-0.28; 0.32]	0.6%
Nunes, V. S. 2022	38	-0.03	0.2890	38	-0.08	0.2910	0.05	[-0.08; 0.18]	2.5%
Theuwissen, E. 2007	43	0.00	0.4900	43	-0.01	0.4750	0.01	[-0.19; 0.21]	1.2%
Seki, Shinji 2003	22	0.08	0.6960	22	0.10	0.8480	-0.01	[-0.47; 0.45]	0.3%
**Random effects model**	**284**			**285**			**0.02**	**[-0.04; 0.08]**	**12.9%**
Heterogeneity: I^2 = 0%, τ^2 = 0, p = 0.99									
**Random effects model**	**895**			**882**			**0.04**	**[0.01; 0.06]**	**100.0%**
Heterogeneity: I^2 = 81%, τ^2 = 0.0015, p < 0.01									
Test for subgroup differences: χ^2_1 = 0.32, df = 1 (p = 0.57)									

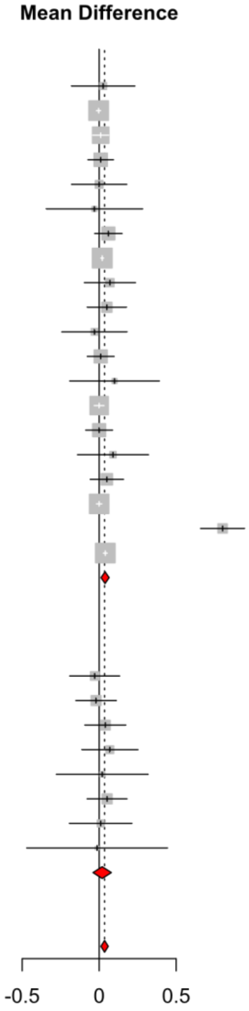

Supplemental Figure 6

Study	Experimental Total	Experimental Mean	Experimental SD	Control Total	Control Mean	Control SD	MD	95%-CI	Weight
**dosage = ≥2**									
Fuentes, F. 2008	30	-0.10	0.2150	30	-0.04	0.2150	-0.06	[-0.17; 0.05]	2.6%
Sola, Rosa 2012	30	-0.06	0.0195	28	0.04	0.0200	-0.10	[-0.11; -0.09]	7.0%
Homma, Y. 2003	33	-0.08	0.0850	34	0.02	0.1150	-0.10	[-0.15; -0.06]	5.3%
Madsen, Martin B. 2007	46	-0.05	0.1200	46	0.00	0.1300	-0.05	[-0.10; 0.00]	5.1%
Temme, E. H. 2002	42	-0.08	0.1700	42	0.01	0.1800	-0.09	[-0.16; -0.02]	3.9%
Castro Cabezas, M. 2006	11	-0.09	0.2120	9	-0.08	0.2350	-0.01	[-0.21; 0.19]	1.0%
de Jong, A. 2008	18	-0.06	0.1400	17	0.01	0.0900	-0.07	[-0.15; 0.01]	3.7%
Jauhiainen, T. 2006	33	-0.13	0.0200	34	-0.10	0.0300	-0.03	[-0.04; -0.02]	6.9%
Hernandez-Mijares, Antonio 2010	31	-0.07	0.1380	24	-0.01	0.1570	-0.06	[-0.14; 0.02]	3.7%
De Jong, A. 2008	15	-0.04	0.1200	11	-0.01	0.0700	-0.03	[-0.10; 0.04]	3.9%
Banuls, Celia 2010	20	-0.03	0.1250	20	-0.03	0.1650	0.00	[-0.09; 0.09]	3.2%
Khandelwal, S. 2009	47	0.01	0.1740	46	0.00	0.1990	0.01	[-0.07; 0.09]	3.8%
Ottestad, I. 2013	21	0.30	0.3000	20	0.40	0.3000	-0.10	[-0.28; 0.08]	1.2%
Chen, S. C. 2009	22	-0.12	0.0300	22	-0.07	0.0300	-0.05	[-0.07; -0.03]	6.8%
Sialvera, T. E. 2012	53	-0.08	0.2400	55	0.00	0.2300	-0.08	[-0.17; 0.01]	3.3%
Theuwissen, E. 2009	14	-0.08	0.1600	14	0.02	0.2250	-0.10	[-0.24; 0.04]	1.7%
Plat, J. 2009	9	-0.07	0.1000	9	-0.02	0.0900	-0.05	[-0.14; 0.04]	3.3%
Judd, J. T. 2002	53	-0.05	0.0174	53	-0.02	0.0174	-0.03	[-0.04; -0.02]	7.0%
Devaraj, S. 2006	36	-0.10	0.2430	36	-0.01	0.2128	-0.09	[-0.20; 0.02]	2.7%
**Random effects model**	**564**			**550**			**-0.06**	**[-0.08; -0.03]**	**76.0%**
Heterogeneity: $I^2 = 87\%$, $\tau^2 = 0.0011$, $p < 0.01$									
**dosage = <2**									
Buyuktuncer, Z. 2013	35	-0.07	0.1960	35	-0.01	0.2010	-0.06	[-0.16; 0.03]	3.1%
Thomsen, A. B. 2004	69	-0.13	0.1900	69	-0.41	0.1900	0.28	[0.22; 0.34]	4.4%
Colgan, H. A. 2004	48	-0.04	0.1050	48	-0.01	0.0900	-0.02	[-0.06; 0.02]	5.8%
Ruiu, G. 2009	15	-0.06	0.3590	15	-0.01	0.3445	-0.05	[-0.30; 0.20]	0.7%
Takeshita, Masao 2008	14	-0.08	0.1752	15	0.01	0.2921	-0.09	[-0.26; 0.08]	1.3%
Theuwissen, E. 2007	43	-0.09	0.2600	43	-0.03	0.2700	-0.06	[-0.17; 0.05]	2.5%
Seki, Shinji 2003	22	-0.08	0.0530	22	-0.04	0.0520	-0.05	[-0.08; -0.02]	6.2%
**Random effects model**	**246**			**247**			**0.00**	**[-0.10; 0.10]**	**24.0%**
Heterogeneity: $I^2 = 93\%$, $\tau^2 = 0.0148$, $p < 0.01$									
**Random effects model**	**810**			**797**			**-0.04**	**[-0.06; -0.02]**	**100.0%**
Heterogeneity: $I^2 = 90\%$, $\tau^2 = 0.0018$, $p < 0.01$									
Test for subgroup differences: $\chi^2_1 = 1.23$, df = 1 ($p = 0.27$)									

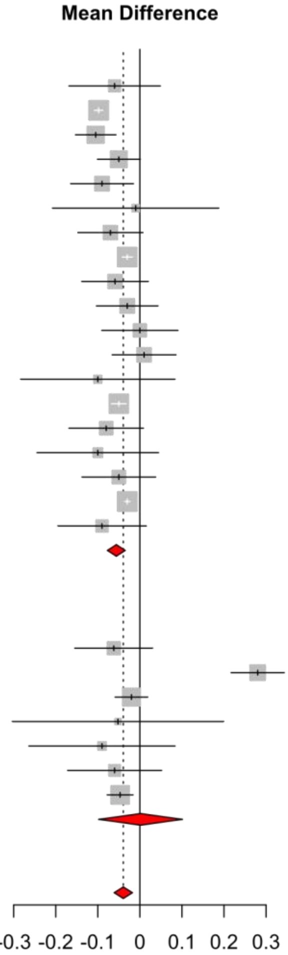

Supplemental Figure 7

Study	Experimental Total	Experimental Mean	Experimental SD	Control Total	Control Mean	Control SD	MD	95%-CI	Weight
**health.condition = unhealthy**									
Fuentes, F. 2008	30	-0.31	1.0600	30	0.00	1.1100	-0.31	[-0.86; 0.24]	1.0%
Buyuktuncer, Z. 2013	35	-0.23	0.6220	35	0.04	0.6550	-0.28	[-0.58; 0.02]	2.7%
Sola, Rosa 2012	30	-0.54	0.1488	28	0.01	0.0767	-0.55	[-0.61; -0.49]	9.5%
Madsen, Martin B. 2007	46	-0.31	0.4500	46	0.01	0.4700	-0.32	[-0.51; -0.13]	4.9%
Thomsen, A. B. 2004	69	-0.88	0.7300	69	-0.39	0.7500	-0.49	[-0.74; -0.24]	3.5%
Temme, E. H. 2002	42	-0.46	0.6720	42	-0.03	0.7260	-0.44	[-0.74; -0.14]	2.7%
Castro Cabezas, M. 2006	11	-0.70	1.8970	9	-0.30	1.2480	-0.40	[-1.78; 0.99]	0.2%
de Jong, A. 2008	18	-0.15	0.4600	17	0.14	0.4100	-0.29	[-0.58; -0.00]	2.8%
Jauhiainen, T. 2006	33	-0.51	0.0600	34	-0.19	0.0600	-0.32	[-0.35; -0.29]	10.4%
Hernandez-Mijares, Antonio 2010	31	-0.45	0.5200	24	-0.01	0.6000	-0.44	[-0.74; -0.14]	2.6%
Banuls, Celia 2010	20	-0.39	0.6880	20	-0.13	0.7800	-0.26	[-0.72; 0.20]	1.3%
Takeshita, Masao 2008	14	-0.28	0.5900	15	-0.02	0.9100	-0.26	[-0.81; 0.29]	0.9%
Sialvera, T. E. 2012	53	-1.01	0.6720	55	0.00	0.6590	-1.01	[-1.26; -0.76]	3.4%
Theuwissen, E. 2009	14	-0.50	0.7000	14	-0.01	0.9200	-0.49	[-1.10; 0.12]	0.8%
Plat, J. 2009	9	-0.75	0.4700	9	0.06	0.4700	-0.81	[-1.24; -0.38]	1.4%
Seki, Shinji 2003	22	-0.06	0.0765	22	0.09	0.6410	-0.14	[-0.41; 0.12]	3.1%
Shaghaghi, Mandana Amir 2014	47	-0.26	0.0700	47	0.20	0.0800	-0.46	[-0.49; -0.43]	10.4%
**Random effects model**	**524**			**516**			**-0.44**	**[-0.52; -0.35]**	**61.5%**
Heterogeneity: $I^2 = 84\%$, $\tau^2 = 0.0129$, $p < 0.01$									
**health.condition = healthy**									
Homma, Y. 2003	33	-0.31	0.4550	34	0.00	0.4650	-0.31	[-0.53; -0.09]	4.0%
De Jong, A. 2008	15	-0.30	0.5600	11	0.04	0.4200	-0.34	[-0.72; 0.04]	1.8%
Colgan, H. A. 2004	48	-0.28	0.7400	48	-0.11	0.6200	-0.17	[-0.44; 0.10]	3.0%
Khandelwal, S. 2009	47	-0.04	0.7200	46	0.12	0.8100	-0.16	[-0.47; 0.15]	2.5%
Ottestad, I. 2013	21	-0.50	0.6000	20	-0.30	0.8000	-0.20	[-0.63; 0.23]	1.4%
Chen, S. C. 2009	22	-0.93	0.1200	22	-0.44	0.1200	-0.49	[-0.56; -0.42]	9.1%
Ruiu, G. 2009	15	-0.21	0.6580	15	0.07	0.5530	-0.28	[-0.72; 0.15]	1.4%
Judd, J. T. 2002	53	-0.80	0.0850	53	-0.41	0.0850	-0.39	[-0.42; -0.36]	10.3%
Nunes, V. S. 2022	38	-0.03	0.8150	38	0.41	0.8030	-0.44	[-0.80; -0.08]	2.0%
Devaraj, S. 2006	36	-0.03	1.1640	36	0.13	0.9700	-0.15	[-0.65; 0.34]	1.2%
Theuwissen, E. 2007	43	-0.48	0.9750	43	-0.23	0.9900	-0.25	[-0.67; 0.17]	1.6%
**Random effects model**	**371**			**366**			**-0.38**	**[-0.44; -0.31]**	**38.5%**
Heterogeneity: $I^2 = 33\%$, $\tau^2 = 0.0028$, $p = 0.13$									
**Random effects model**	**895**			**882**			**-0.40**	**[-0.46; -0.35]**	**100.0%**
Heterogeneity: $I^2 = 77\%$, $\tau^2 = 0.0077$, $p < 0.01$									
Test for subgroup differences: $\chi^2_1 = 1.21$, df = 1 ($p = 0.27$)									

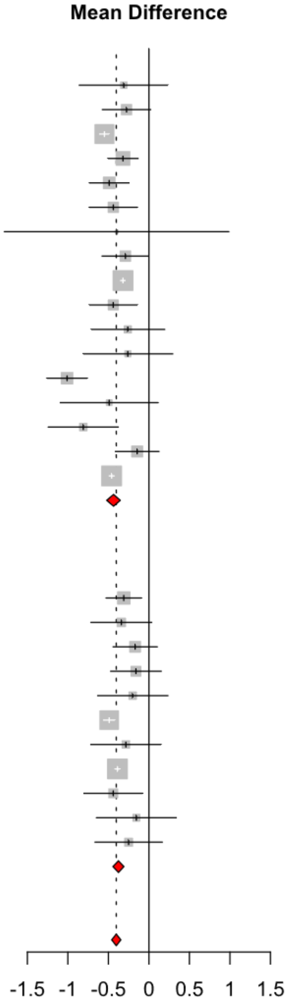

Supplemental Figure 8

Study	Experimental Total	Experimental Mean	Experimental SD	Control Total	Control Mean	Control SD	MD	95%-CI	Weight
**health.condition = unhealthy**									
Fuentes, F. 2008	30	-0.28	1.0470	30	0.03	1.0470	-0.31	[-0.84; 0.22]	2.5%
Buyuktuncer, Z. 2013	35	-0.26	0.6580	35	-0.01	0.5160	-0.25	[-0.53; 0.02]	3.9%
Sola, Rosa 2012	30	-0.47	0.0825	28	0.07	0.0850	-0.54	[-0.58; -0.50]	5.0%
Madsen, Martin B. 2007	46	-0.34	0.3900	46	-0.05	0.4100	-0.29	[-0.45; -0.13]	4.6%
Thomsen, A. B. 2004	69	-0.60	0.7600	69	-0.16	0.8000	-0.44	[-0.70; -0.18]	4.0%
Temme, E. H. 2002	42	-0.41	0.6080	42	0.00	0.6460	-0.41	[-0.68; -0.14]	4.0%
Castro Cabezas, M. 2006	11	-0.72	1.1970	9	-0.40	1.3980	-0.33	[-1.48; 0.83]	0.8%
de Jong, A. 2008	18	-0.19	0.4200	17	0.09	0.3800	-0.28	[-0.55; -0.01]	4.0%
Jauhiainen, T. 2006	33	-0.48	0.0600	34	-0.12	0.0600	-0.36	[-0.39; -0.33]	5.1%
Hernandez-Mijares, Antonio 2010	31	-0.43	0.4500	24	0.00	0.5100	-0.43	[-0.69; -0.17]	4.1%
Banuls, Celia 2010	20	-0.42	0.4800	20	-0.08	0.7000	-0.34	[-0.71; 0.03]	3.3%
Takeshita, Masao 2008	14	-0.21	0.5200	15	0.03	0.9000	-0.24	[-0.77; 0.29]	2.5%
Sialvera, T. E. 2012	53	-0.88	0.5690	55	0.05	0.6080	-0.93	[-1.15; -0.71]	4.3%
Theuwissen, E. 2009	14	-0.48	0.6800	14	-0.01	0.9300	-0.47	[-1.07; 0.13]	2.1%
Seki, Shinji 2003	22	-0.25	0.4600	22	0.15	0.4340	-0.40	[-0.66; -0.13]	4.0%
Shaghaghi, Mandana Amir 2014	47	-0.27	0.0600	47	0.17	0.0800	-0.44	[-0.47; -0.41]	5.1%
**Random effects model**	**515**			**507**			**-0.43**	**[-0.50; -0.36]**	**59.3%**
Heterogeneity: $I^2 = 80\%$, $\tau^2 = 0.0073$, $p < 0.01$									
**health.condition = healthy**									
Homma, Y. 2003	33	-0.38	0.2800	34	-0.04	0.3600	-0.34	[-0.49; -0.19]	4.7%
De Jong, A. 2008	15	-0.27	0.3900	11	0.01	0.3700	-0.28	[-0.57; 0.01]	3.8%
Colgan, H. A. 2004	48	-0.28	0.9200	48	-0.14	0.6700	-0.14	[-0.46; 0.18]	3.6%
Khandelwal, S. 2009	47	0.01	0.7500	46	0.12	0.6800	-0.11	[-0.40; 0.18]	3.8%
Ottestad, I. 2013	21	-0.30	0.7000	20	-0.20	0.8000	-0.10	[-0.56; 0.36]	2.8%
Chen, S. C. 2009	22	-0.73	0.0850	22	-0.30	0.0850	-0.43	[-0.48; -0.38]	5.0%
Ruiu, G. 2009	15	-0.30	0.9130	15	0.08	0.7950	-0.38	[-0.99; 0.23]	2.1%
Judd, J. T. 2002	53	-0.62	0.0649	53	0.28	0.0649	-0.90	[-0.93; -0.88]	5.1%
Nunes, V. S. 2022	38	0.10	0.8200	38	0.46	0.8140	-0.36	[-0.73; 0.01]	3.4%
Devaraj, S. 2006	36	-0.13	0.8020	36	0.00	0.9310	-0.13	[-0.53; 0.27]	3.1%
Theuwissen, E. 2007	43	-0.42	0.9300	43	-0.22	0.9350	-0.20	[-0.59; 0.19]	3.2%
**Random effects model**	**371**			**366**			**-0.32**	**[-0.55; -0.09]**	**40.7%**
Heterogeneity: $I^2 = 97\%$, $\tau^2 = 0.1245$, $p < 0.01$									
**Random effects model**	**886**			**873**			**-0.38**	**[-0.50; -0.27]**	**100.0%**
Heterogeneity: $I^2 = 98\%$, $\tau^2 = 0.0683$, $p < 0.01$									
Test for subgroup differences: $\chi^2_1 = 0.77$, df = 1 ($p = 0.38$)									

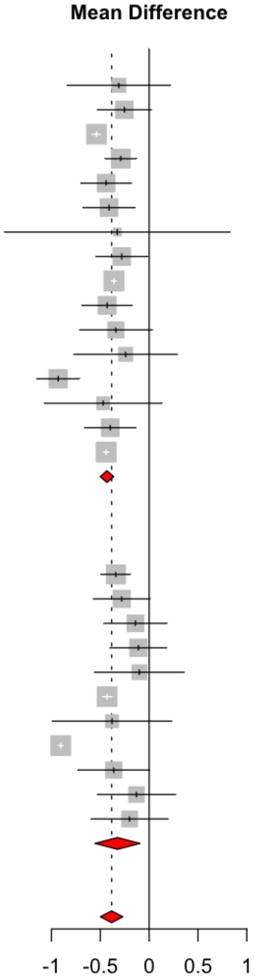

Supplemental Figure 9

A

Study	Experimental Total	Experimental Mean	Experimental SD	Control Total	Control Mean	Control SD	MD	95%-CI	Weight
**health.condition = unhealthy**									
Fuentes, F. 2008	30	-0.01	0.2550	30	-0.04	0.2700	0.03	[-0.10; 0.16]	0.3%
Buyuktuncer, Z. 2013	35	0.02	0.2990	35	-0.02	0.1990	0.03	[-0.09; 0.15]	0.4%
Sola, Rosa 2012	30	0.01	0.0230	28	0.02	0.0240	-0.01	[-0.02; 0.00]	39.0%
Madsen, Martin B. 2007	46	0.00	0.1200	46	0.01	0.1200	-0.01	[-0.06; 0.04]	2.4%
Temme, E. H. 2002	42	-0.01	0.2700	42	0.00	0.2800	-0.01	[-0.13; 0.11]	0.4%
Castro Cabezas, M. 2006	11	-0.10	0.2295	9	-0.04	0.2654	-0.06	[-0.28; 0.16]	0.1%
de Jong, A. 2008	18	0.06	0.1000	17	0.06	0.1300	0.00	[-0.08; 0.08]	1.0%
Hernandez-Mijares, Antonio 2010	31	0.06	0.2410	24	-0.03	0.2410	0.09	[-0.04; 0.22]	0.3%
Sialvera, T. E. 2012	53	0.00	0.1800	55	0.00	0.2200	0.00	[-0.08; 0.08]	1.0%
Theuwissen, E. 2009	14	-0.01	0.1900	14	0.00	0.3450	-0.01	[-0.22; 0.20]	0.1%
Plat, J. 2009	9	-0.03	0.0500	9	0.00	0.0300	-0.03	[-0.07; 0.01]	3.9%
Seki, Shinji 2003	22	0.01	0.0690	22	-0.02	0.0580	0.03	[-0.01; 0.07]	4.0%
**Random effects model**	**341**			**331**			**-0.01**	**[-0.02; 0.00]**	**53.1%**
Heterogeneity: $I^2 = 0\%$, $\tau^2 = 0$, $p = 0.60$									
**health.condition = healthy**									
Homma, Y. 2003	33	0.02	0.0945	34	0.02	0.1160	-0.00	[-0.06; 0.05]	2.2%
De Jong, A. 2008	15	0.01	0.1000	11	-0.02	0.1500	0.03	[-0.07; 0.13]	0.5%
Khandelwal, S. 2009	47	0.00	0.2340	46	-0.02	0.1940	0.02	[-0.07; 0.11]	0.8%
Ottestad, I. 2013	21	-0.30	0.2000	20	-0.40	0.2000	0.10	[-0.02; 0.22]	0.4%
Chen, S. C. 2009	22	-0.11	0.0400	22	-0.11	0.0400	0.00	[-0.02; 0.02]	10.2%
Ruiu, G. 2009	15	0.03	0.0967	15	-0.02	0.1013	0.05	[-0.02; 0.12]	1.1%
Judd, J. T. 2002	53	-0.07	0.0355	53	-0.06	0.0355	-0.01	[-0.02; 0.00]	31.3%
Theuwissen, E. 2007	43	-0.05	0.3150	43	-0.02	0.3250	-0.03	[-0.17; 0.11]	0.3%
**Random effects model**	**249**			**244**			**-0.00**	**[-0.02; 0.01]**	**46.9%**
Heterogeneity: $I^2 = 0\%$, $\tau^2 = 0$, $p = 0.46$									
**Random effects model**	**590**			**575**			**-0.01**	**[-0.01; 0.00]**	**100.0%**
Heterogeneity: $I^2 = 0\%$, $\tau^2 = 0$, $p = 0.64$									
Test for subgroup differences: $\chi^2_1 = 0.26$, df = 1 ($p = 0.61$)									

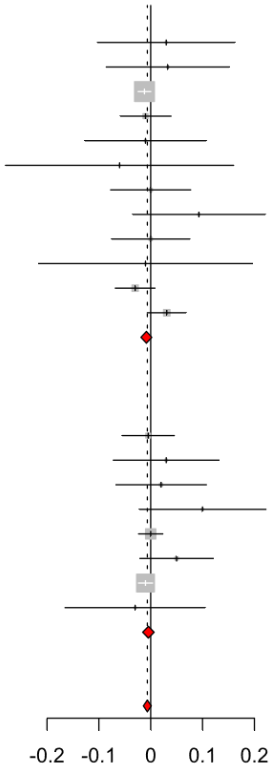

B

Study	Experimental Total	Experimental Mean	Experimental SD	Control Total	Control Mean	Control SD	MD	95%-CI	Weight
**health.condition = unhealthy**									
Fuentes, F. 2008	30	-0.10	0.2150	30	-0.04	0.2150	-0.06	[-0.17; 0.05]	2.6%
Buyuktuncer, Z. 2013	35	-0.07	0.1960	35	-0.01	0.2010	-0.06	[-0.16; 0.03]	3.1%
Sola, Rosa 2012	30	-0.06	0.0195	28	0.04	0.0200	-0.10	[-0.11; -0.09]	7.0%
Madsen, Martin B. 2007	46	-0.05	0.1200	46	0.00	0.1300	-0.05	[-0.10; 0.00]	5.1%
Thomsen, A. B. 2004	69	-0.13	0.1900	69	-0.41	0.1900	0.28	[0.22; 0.34]	4.4%
Temme, E. H. 2002	42	-0.08	0.1700	42	0.01	0.1800	-0.09	[-0.16; -0.02]	3.9%
Castro Cabezas, M. 2006	11	-0.09	0.2120	9	-0.08	0.2350	-0.01	[-0.21; 0.19]	1.0%
de Jong, A. 2008	18	-0.06	0.1400	17	0.01	0.0900	-0.07	[-0.15; 0.01]	3.7%
Jauhiainen, T. 2006	33	-0.13	0.0200	34	-0.10	0.0300	-0.03	[-0.04; -0.02]	6.9%
Hernandez-Mijares, Antonio 2010	31	-0.07	0.1380	24	-0.01	0.1570	-0.06	[-0.14; 0.02]	3.7%
Banuls, Celia 2010	20	-0.03	0.1250	20	-0.03	0.1650	0.00	[-0.09; 0.09]	3.2%
Takeshita, Masao 2008	14	-0.08	0.1752	15	0.01	0.2921	-0.09	[-0.26; 0.08]	1.3%
Sialvera, T. E. 2012	53	-0.08	0.2400	55	0.00	0.2300	-0.08	[-0.17; 0.01]	3.3%
Theuwissen, E. 2009	14	-0.08	0.1600	14	0.02	0.2250	-0.10	[-0.24; 0.04]	1.7%
Plat, J. 2009	9	-0.07	0.1000	9	-0.02	0.0900	-0.05	[-0.14; 0.04]	3.3%
Seki, Shinji 2003	22	-0.08	0.0530	22	-0.04	0.0520	-0.05	[-0.08; -0.02]	6.2%
**Random effects model**	**477**			**469**			**-0.04**	**[-0.07; 0.00]**	**60.4%**
Heterogeneity: $I^2 = 92\%$, $\tau^2 = 0.0038$, $p < 0.01$									
**health.condition = healthy**									
Homma, Y. 2003	33	-0.08	0.0850	34	0.02	0.1150	-0.10	[-0.15; -0.06]	5.3%
De Jong, A. 2008	15	-0.04	0.1200	11	-0.01	0.0700	-0.03	[-0.10; 0.04]	3.9%
Colgan, H. A. 2004	48	-0.04	0.1050	48	-0.01	0.0900	-0.02	[-0.06; 0.02]	5.8%
Khandelwal, S. 2009	47	0.01	0.1740	46	0.00	0.1990	0.01	[-0.07; 0.09]	3.8%
Ottestad, I. 2013	21	0.30	0.3000	20	0.40	0.3000	-0.10	[-0.28; 0.08]	1.2%
Chen, S. C. 2009	22	-0.12	0.0300	22	-0.07	0.0300	-0.05	[-0.07; -0.03]	6.8%
Ruiu, G. 2009	15	-0.06	0.3590	15	-0.01	0.3445	-0.05	[-0.30; 0.20]	0.7%
Judd, J. T. 2002	53	-0.05	0.0174	53	-0.02	0.0174	-0.03	[-0.04; -0.02]	7.0%
Devaraj, S. 2006	36	-0.10	0.2430	36	-0.01	0.2128	-0.09	[-0.20; 0.02]	2.7%
Theuwissen, E. 2007	43	-0.09	0.2600	43	-0.03	0.2700	-0.06	[-0.17; 0.05]	2.5%
**Random effects model**	**333**			**328**			**-0.04**	**[-0.06; -0.02]**	**39.6%**
Heterogeneity: $I^2 = 45\%$, $\tau^2 = 0.0002$, $p = 0.06$									
**Random effects model**	**810**			**797**			**-0.04**	**[-0.06; -0.02]**	**100.0%**
Heterogeneity: $I^2 = 90\%$, $\tau^2 = 0.0018$, $p < 0.01$									
Test for subgroup differences: $\chi^2_1 = 0.13$, df = 1 ($p = 0.72$)									

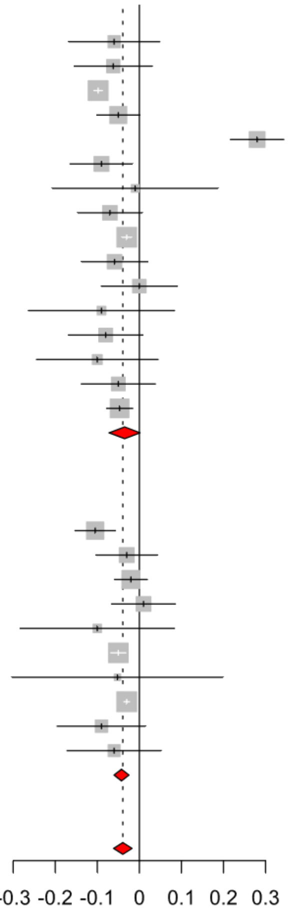

Supplemental Figure 10

Study	Experimental Total	Experimental Mean	Experimental SD	Control Total	Control Mean	Control SD	MD	95%-CI	Weight
**duration = ≤4w**									
Fuentes, F. 2008	30	-0.31	1.0600	30	0.00	1.1100	-0.31	[-0.86; 0.24]	1.0%
Buyuktuncer, Z. 2013	35	-0.23	0.6220	35	0.04	0.6550	-0.28	[-0.58; 0.02]	2.7%
Sola, Rosa 2012	30	-0.54	0.1488	28	0.01	0.0767	-0.55	[-0.61; -0.49]	9.5%
Homma, Y. 2003	33	-0.31	0.4550	34	0.00	0.4650	-0.31	[-0.53; -0.09]	4.0%
Madsen, Martin B. 2007	46	-0.31	0.4500	46	0.01	0.4700	-0.32	[-0.51; -0.13]	4.9%
Thomsen, A. B. 2004	69	-0.88	0.7300	69	-0.39	0.7500	-0.49	[-0.74; -0.24]	3.5%
Temme, E. H. 2002	42	-0.46	0.6720	42	-0.03	0.7260	-0.44	[-0.74; -0.14]	2.7%
Colgan, H. A. 2004	48	-0.28	0.7400	48	-0.11	0.6200	-0.17	[-0.44; 0.10]	3.0%
Khandelwal, S. 2009	47	-0.04	0.7200	46	0.12	0.8100	-0.16	[-0.47; 0.15]	2.5%
Ottestad, I. 2013	21	-0.50	0.6000	20	-0.30	0.8000	-0.20	[-0.63; 0.23]	1.4%
Chen, S. C. 2009	22	-0.93	0.1200	22	-0.44	0.1200	-0.49	[-0.56; -0.42]	9.1%
Ruiu, G. 2009	15	-0.21	0.6580	15	0.07	0.5530	-0.28	[-0.72; 0.15]	1.4%
Theuwissen, E. 2009	14	-0.50	0.7000	14	-0.01	0.9200	-0.49	[-1.10; 0.12]	0.8%
Judd, J. T. 2002	53	-0.80	0.0850	53	-0.41	0.0850	-0.39	[-0.42; -0.36]	10.3%
Nunes, V. S. 2022	38	-0.03	0.8150	38	0.41	0.8030	-0.44	[-0.80; -0.08]	2.0%
Theuwissen, E. 2007	43	-0.48	0.9750	43	-0.23	0.9900	-0.25	[-0.67; 0.17]	1.6%
Seki, Shinji 2003	22	-0.06	0.0765	22	0.09	0.6410	-0.14	[-0.41; 0.12]	3.1%
Shaghaghi, Mandana Amir 2014	47	-0.26	0.0700	47	0.20	0.0800	-0.46	[-0.49; -0.43]	10.4%
**Random effects model**	**655**			**652**			**-0.41**	**[-0.46; -0.36]**	**73.9%**
Heterogeneity: $I^2 = 62\%$, $\tau^2 = 0.0038$, $p < 0.01$									
**duration = >4w**									
Castro Cabezas, M. 2006	11	-0.70	1.8970	9	-0.30	1.2480	-0.40	[-1.78; 0.99]	0.2%
de Jong, A. 2008	18	-0.15	0.4600	17	0.14	0.4100	-0.29	[-0.58; -0.00]	2.8%
Jauhiainen, T. 2006	33	-0.51	0.0600	34	-0.19	0.0600	-0.32	[-0.35; -0.29]	10.4%
Hernandez-Mijares, Antonio 2010	31	-0.45	0.5200	24	-0.01	0.6000	-0.44	[-0.74; -0.14]	2.6%
De Jong, A. 2008	15	-0.30	0.5600	11	0.04	0.4200	-0.34	[-0.72; 0.04]	1.8%
Banuls, Celia 2010	20	-0.39	0.6880	20	-0.13	0.7800	-0.26	[-0.72; 0.20]	1.3%
Takeshita, Masao 2008	14	-0.28	0.5900	15	-0.02	0.9100	-0.26	[-0.81; 0.29]	0.9%
Sialvera, T. E. 2012	53	-1.01	0.6720	55	0.00	0.6590	-1.01	[-1.26; -0.76]	3.4%
Plat, J. 2009	9	-0.75	0.4700	9	0.06	0.4700	-0.81	[-1.24; -0.38]	1.4%
Devaraj, S. 2006	36	-0.03	1.1640	36	0.13	0.9700	-0.15	[-0.65; 0.34]	1.2%
**Random effects model**	**240**			**230**			**-0.45**	**[-0.64; -0.26]**	**26.1%**
Heterogeneity: $I^2 = 74\%$, $\tau^2 = 0.0536$, $p < 0.01$									
**Random effects model**	**895**			**882**			**-0.40**	**[-0.46; -0.35]**	**100.0%**
Heterogeneity: $I^2 = 77\%$, $\tau^2 = 0.0077$, $p < 0.01$									
Test for subgroup differences: $\chi^2_1 = 0.15$, df = 1 ($p = 0.70$)									

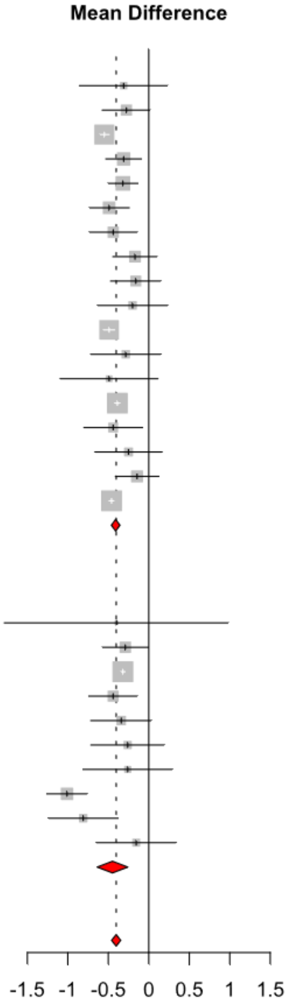

Supplemental Figure 11

Study	Experimental Total	Experimental Mean	Experimental SD	Control Total	Control Mean	Control SD	MD	95%-CI	Weight
**duration = ≤4w**									
Fuentes, F. 2008	30	-0.28	1.0470	30	0.03	1.0470	-0.31	[-0.84; 0.22]	2.5%
Buyuktuncer, Z. 2013	35	-0.26	0.6580	35	-0.01	0.5160	-0.25	[-0.53; 0.02]	3.9%
Sola, Rosa 2012	30	-0.47	0.0825	28	0.07	0.0850	-0.54	[-0.58; -0.50]	5.0%
Homma, Y. 2003	33	-0.38	0.2800	34	-0.04	0.3600	-0.34	[-0.49; -0.19]	4.7%
Madsen, Martin B. 2007	46	-0.34	0.3900	46	-0.05	0.4100	-0.29	[-0.45; -0.13]	4.6%
Thomsen, A. B. 2004	69	-0.60	0.7600	69	-0.16	0.8000	-0.44	[-0.70; -0.18]	4.0%
Temme, E. H. 2002	42	-0.41	0.6080	42	0.00	0.6460	-0.41	[-0.68; -0.14]	4.0%
Colgan, H. A. 2004	48	-0.28	0.9200	48	-0.14	0.6700	-0.14	[-0.46; 0.18]	3.6%
Khandelwal, S. 2009	47	0.01	0.7500	46	0.12	0.6800	-0.11	[-0.40; 0.18]	3.8%
Ottestad, I. 2013	21	-0.30	0.7000	20	-0.20	0.8000	-0.10	[-0.56; 0.36]	2.8%
Chen, S. C. 2009	22	-0.73	0.0850	22	-0.30	0.0850	-0.43	[-0.48; -0.38]	5.0%
Ruiu, G. 2009	15	-0.30	0.9130	15	0.08	0.7950	-0.38	[-0.99; 0.23]	2.1%
Theuwissen, E. 2009	14	-0.48	0.6800	14	-0.01	0.9300	-0.47	[-1.07; 0.13]	2.1%
Judd, J. T. 2002	53	-0.62	0.0649	53	0.28	0.0649	-0.90	[-0.93; -0.88]	5.1%
Nunes, V. S. 2022	38	0.10	0.8200	38	0.46	0.8140	-0.36	[-0.73; 0.01]	3.4%
Theuwissen, E. 2007	43	-0.42	0.9300	43	-0.22	0.9350	-0.20	[-0.59; 0.19]	3.2%
Seki, Shinji 2003	22	-0.25	0.4600	22	0.15	0.4340	-0.40	[-0.66; -0.13]	4.0%
Shaghaghi, Mandana Amir 2014	47	-0.27	0.0600	47	0.17	0.0800	-0.44	[-0.47; -0.41]	5.1%
**Random effects model**	**655**			**652**			**-0.38**	**[-0.52; -0.24]**	**69.0%**
Heterogeneity: $I^2 = 98\%$, $\tau^2 = 0.0726$, $p < 0.01$									
**duration = >4w**									
Castro Cabezas, M. 2006	11	-0.72	1.1970	9	-0.40	1.3980	-0.33	[-1.48; 0.83]	0.8%
de Jong, A. 2008	18	-0.19	0.4200	17	0.09	0.3800	-0.28	[-0.55; -0.01]	4.0%
Jauhiainen, T. 2006	33	-0.48	0.0600	34	-0.12	0.0600	-0.36	[-0.39; -0.33]	5.1%
Hernandez-Mijares, Antonio 2010	31	-0.43	0.4500	24	0.00	0.5100	-0.43	[-0.69; -0.17]	4.1%
De Jong, A. 2008	15	-0.27	0.3900	11	0.01	0.3700	-0.28	[-0.57; 0.01]	3.8%
Banuls, Celia 2010	20	-0.42	0.4800	20	-0.08	0.7000	-0.34	[-0.71; 0.03]	3.3%
Takeshita, Masao 2008	14	-0.21	0.5200	15	0.03	0.9000	-0.24	[-0.77; 0.29]	2.5%
Sialvera, T. E. 2012	53	-0.88	0.5690	55	0.05	0.6080	-0.93	[-1.15; -0.71]	4.3%
Devaraj, S. 2006	36	-0.13	0.8020	36	0.00	0.9310	-0.13	[-0.53; 0.27]	3.1%
**Random effects model**	**231**			**221**			**-0.40**	**[-0.56; -0.24]**	**31.0%**
Heterogeneity: $I^2 = 71\%$, $\tau^2 = 0.0335$, $p < 0.01$									
**Random effects model**	**886**			**873**			**-0.38**	**[-0.50; -0.27]**	**100.0%**
Heterogeneity: $I^2 = 98\%$, $\tau^2 = 0.0683$, $p < 0.01$									
Test for subgroup differences: $\chi^2_1 = 0.04$, df = 1 ($p = 0.84$)									

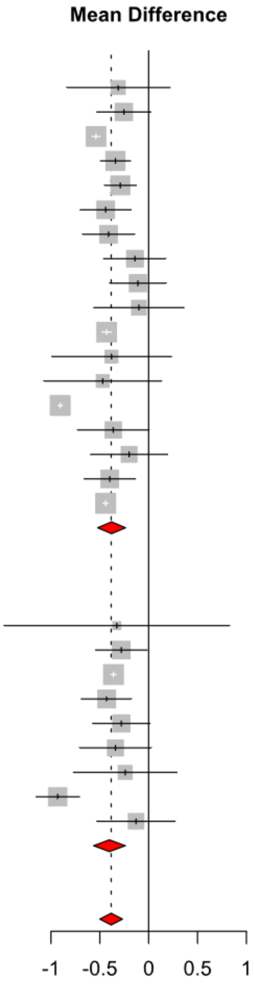

Supplemental Figure 12

Study	Experimental Total	Experimental Mean	Experimental SD	Control Total	Control Mean	Control SD	MD	95%-CI	Weight
**duration = ≤4w**									
Fuentes, F. 2008	30	0.00	0.3880	30	-0.03	0.4270	0.03	[-0.18; 0.23]	1.2%
Buyuktuncer, Z. 2013	35	-0.01	0.3900	35	0.02	0.2980	-0.03	[-0.19; 0.13]	1.8%
Sola, Rosa 2012	30	-0.05	0.0278	28	-0.05	0.0290	-0.00	[-0.02; 0.01]	9.7%
Homma, Y. 2003	33	0.01	0.1100	34	0.00	0.1100	0.01	[-0.04; 0.06]	6.8%
Madsen, Martin B. 2007	46	0.12	0.2100	46	0.11	0.2000	0.01	[-0.07; 0.09]	4.5%
Thomsen, A. B. 2004	69	-0.02	0.3900	69	0.00	0.4000	-0.02	[-0.15; 0.11]	2.5%
Temme, E. H. 2002	42	0.00	0.4140	42	0.00	0.4270	0.00	[-0.18; 0.18]	1.5%
Colgan, H. A. 2004	48	-0.01	0.3400	48	-0.05	0.3300	0.04	[-0.09; 0.17]	2.4%
Khandelwal, S. 2009	47	-0.01	0.1900	46	-0.02	0.2400	0.01	[-0.08; 0.10]	4.3%
Ottestad, I. 2013	21	-0.10	0.4500	20	-0.20	0.5000	0.10	[-0.19; 0.39]	0.6%
Chen, S. C. 2009	22	-0.09	0.0550	22	-0.09	0.0550	0.00	[-0.03; 0.03]	8.5%
Ruiu, G. 2009	15	0.05	0.2780	15	-0.02	0.2310	0.07	[-0.11; 0.25]	1.5%
Theuwissen, E. 2009	14	-0.02	0.1750	14	-0.11	0.4050	0.09	[-0.14; 0.32]	1.0%
Judd, J. T. 2002	53	-0.04	0.0480	53	-0.04	0.0480	0.00	[-0.02; 0.02]	9.5%
Nunes, V. S. 2022	38	-0.03	0.2890	38	-0.08	0.2910	0.05	[-0.08; 0.18]	2.5%
Theuwissen, E. 2007	43	0.00	0.4900	43	-0.01	0.4750	0.01	[-0.19; 0.21]	1.2%
Seki, Shinji 2003	22	0.08	0.6960	22	0.10	0.8480	-0.01	[-0.47; 0.45]	0.3%
Shaghaghi, Mandana Amir 2014	47	0.03	0.0200	47	-0.01	0.0400	0.04	[0.03; 0.05]	9.7%
**Random effects model**	**655**			**652**			**0.01**	**[-0.00; 0.03]**	**69.5%**
Heterogeneity: $I^2 = 35\%$, $\tau^2 = 0.0002$, $p = 0.07$									
**duration = >4w**									
Castro Cabezas, M. 2006	11	0.00	0.2950	9	0.03	0.4000	-0.03	[-0.35; 0.28]	0.6%
de Jong, A. 2008	18	0.10	0.1300	17	0.04	0.1400	0.06	[-0.03; 0.15]	4.2%
Jauhiainen, T. 2006	33	-0.01	0.0200	34	-0.03	0.0200	0.02	[0.01; 0.03]	9.9%
Hernandez-Mijares, Antonio 2010	31	0.05	0.3700	24	-0.02	0.2600	0.07	[-0.10; 0.24]	1.7%
De Jong, A. 2008	15	0.04	0.1600	11	-0.01	0.1700	0.05	[-0.08; 0.18]	2.6%
Banuls, Celia 2010	20	-0.05	0.3900	20	-0.02	0.2900	-0.03	[-0.24; 0.18]	1.1%
Takeshita, Masao 2008	14	-0.05	0.4050	15	-0.07	0.4150	0.02	[-0.28; 0.32]	0.6%
Sialvera, T. E. 2012	53	0.00	0.2330	55	0.00	0.2330	0.00	[-0.09; 0.09]	4.3%
Plat, J. 2009	9	-0.02	0.1400	9	-0.07	0.0900	0.05	[-0.06; 0.16]	3.3%
Devaraj, S. 2006	36	0.78	0.2970	36	-0.03	0.3230	0.80	[0.66; 0.95]	2.2%
**Random effects model**	**240**			**230**			**0.11**	**[-0.01; 0.22]**	**30.5%**
Heterogeneity: $I^2 = 92\%$, $\tau^2 = 0.0271$, $p < 0.01$									
**Random effects model**	**895**			**882**			**0.04**	**[0.01; 0.06]**	**100.0%**
Heterogeneity: $I^2 = 81\%$, $\tau^2 = 0.0015$, $p < 0.01$									
Test for subgroup differences: $\chi^2_1 = 2.66$, df = 1 ($p = 0.10$)									

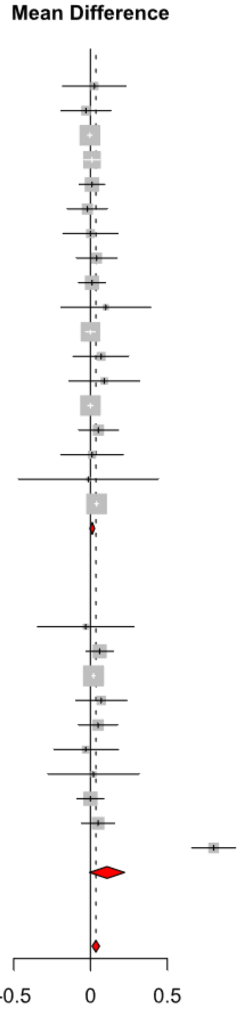

Supplemental Figure 13

A

Study	Experimental Total	Experimental Mean	Experimental SD	Control Total	Control Mean	Control SD	MD	95%-CI	Weight
**duration = ≤4w**									
Fuentes, F. 2008	30	-0.01	0.2550	30	-0.04	0.2700	0.03	[-0.10; 0.16]	0.3%
Buyuktuncer, Z. 2013	35	0.02	0.2990	35	-0.02	0.1990	0.03	[-0.09; 0.15]	0.4%
Sola, Rosa 2012	30	0.01	0.0230	28	0.02	0.0240	-0.01	[-0.02; 0.00]	39.0%
Homma, Y. 2003	33	0.02	0.0945	34	0.02	0.1160	-0.00	[-0.06; 0.05]	2.2%
Madsen, Martin B. 2007	46	0.00	0.1200	46	0.01	0.1200	-0.01	[-0.06; 0.04]	2.4%
Temme, E. H. 2002	42	-0.01	0.2700	42	0.00	0.2800	-0.01	[-0.13; 0.11]	0.4%
Khandelwal, S. 2009	47	0.00	0.2340	46	-0.02	0.1940	0.02	[-0.07; 0.11]	0.8%
Ottestad, I. 2013	21	-0.30	0.2000	20	-0.40	0.2000	0.10	[-0.02; 0.22]	0.4%
Chen, S. C. 2009	22	-0.11	0.0400	22	-0.11	0.0400	0.00	[-0.02; 0.02]	10.2%
Ruiu, G. 2009	15	0.03	0.0967	15	-0.02	0.1013	0.05	[-0.02; 0.12]	1.1%
Theuwissen, E. 2009	14	-0.01	0.1900	14	0.00	0.3450	-0.01	[-0.22; 0.20]	0.1%
Judd, J. T. 2002	53	-0.07	0.0355	53	-0.06	0.0355	-0.01	[-0.02; 0.00]	31.3%
Theuwissen, E. 2007	43	-0.05	0.3150	43	-0.02	0.3250	-0.03	[-0.17; 0.11]	0.3%
Seki, Shinji 2003	22	0.01	0.0690	22	-0.02	0.0580	0.03	[-0.01; 0.07]	4.0%
**Random effects model**	**453**			**450**			**-0.01**	**[-0.01; 0.00]**	**93.1%**
Heterogeneity: $I^2 = 0\%$, $\tau^2 = 0$, $p = 0.55$									
**duration = >4w**									
Castro Cabezas, M. 2006	11	-0.10	0.2295	9	-0.04	0.2654	-0.06	[-0.28; 0.16]	0.1%
de Jong, A. 2008	18	0.06	0.1000	17	0.06	0.1300	0.00	[-0.08; 0.08]	1.0%
Hernandez-Mijares, Antonio 2010	31	0.06	0.2410	24	-0.03	0.2410	0.09	[-0.04; 0.22]	0.3%
De Jong, A. 2008	15	0.01	0.1000	11	-0.02	0.1500	0.03	[-0.07; 0.13]	0.5%
Sialvera, T. E. 2012	53	0.00	0.1800	55	0.00	0.2200	0.00	[-0.08; 0.08]	1.0%
Plat, J. 2009	9	-0.03	0.0500	9	0.00	0.0300	-0.03	[-0.07; 0.01]	3.9%
**Random effects model**	**137**			**125**			**-0.01**	**[-0.04; 0.02]**	**6.9%**
Heterogeneity: $I^2 = 0\%$, $\tau^2 = 0$, $p = 0.49$									
**Random effects model**	**590**			**575**			**-0.01**	**[-0.01; 0.00]**	**100.0%**
Heterogeneity: $I^2 = 0\%$, $\tau^2 = 0$, $p = 0.64$									
Test for subgroup differences: $\chi^2_1 = 0.11$, df = 1 ($p = 0.75$)									

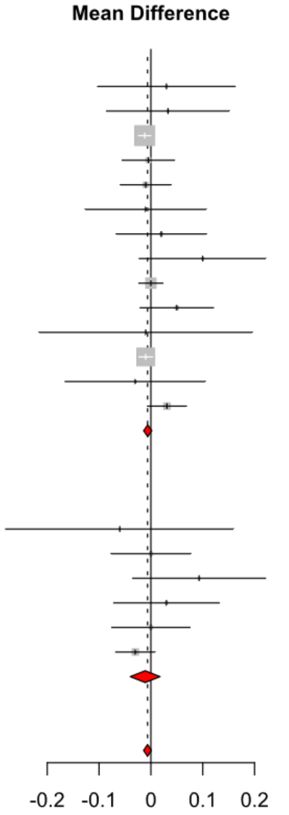

B

Study	Experimental Total	Experimental Mean	Experimental SD	Control Total	Control Mean	Control SD	MD	95%-CI	Weight
**duration = ≤4w**									
Fuentes, F. 2008	30	-0.10	0.2150	30	-0.04	0.2150	-0.06	[-0.17; 0.05]	2.6%
Buyuktuncer, Z. 2013	35	-0.07	0.1960	35	-0.01	0.2010	-0.06	[-0.16; 0.03]	3.1%
Sola, Rosa 2012	30	-0.06	0.0195	28	0.04	0.0200	-0.10	[-0.11; -0.09]	7.0%
Homma, Y. 2003	33	-0.08	0.0850	34	0.02	0.1150	-0.10	[-0.15; -0.06]	5.3%
Madsen, Martin B. 2007	46	-0.05	0.1200	46	0.00	0.1300	-0.05	[-0.10; 0.00]	5.1%
Thomsen, A. B. 2004	69	-0.13	0.1900	69	-0.41	0.1900	0.28	[0.22; 0.34]	4.4%
Temme, E. H. 2002	42	-0.08	0.1700	42	0.01	0.1800	-0.09	[-0.16; -0.02]	3.9%
Colgan, H. A. 2004	48	-0.04	0.1050	48	-0.01	0.0900	-0.02	[-0.06; 0.02]	5.8%
Khandelwal, S. 2009	47	0.01	0.1740	46	0.00	0.1990	0.01	[-0.07; 0.09]	3.8%
Ottestad, I. 2013	21	0.30	0.3000	20	0.40	0.3000	-0.10	[-0.28; 0.08]	1.2%
Chen, S. C. 2009	22	-0.12	0.0300	22	-0.07	0.0300	-0.05	[-0.07; -0.03]	6.8%
Ruiu, G. 2009	15	-0.06	0.3590	15	-0.01	0.3445	-0.05	[-0.30; 0.20]	0.7%
Theuwissen, E. 2009	14	-0.08	0.1600	14	0.02	0.2250	-0.10	[-0.24; 0.04]	1.7%
Judd, J. T. 2002	53	-0.05	0.0174	53	-0.02	0.0174	-0.03	[-0.04; -0.02]	7.0%
Theuwissen, E. 2007	43	-0.09	0.2600	43	-0.03	0.2700	-0.06	[-0.17; 0.05]	2.5%
Seki, Shinji 2003	22	-0.08	0.0530	22	-0.04	0.0520	-0.05	[-0.08; -0.02]	6.2%
**Random effects model**	**570**			**567**			**-0.03**	**[-0.06; -0.00]**	**66.9%**
Heterogeneity: $I^2 = 94\%$, $\tau^2 = 0.0025$, $p < 0.01$									
**duration = >4w**									
Castro Cabezas, M. 2006	11	-0.09	0.2120	9	-0.08	0.2350	-0.01	[-0.21; 0.19]	1.0%
de Jong, A. 2008	18	-0.06	0.1400	17	0.01	0.0900	-0.07	[-0.15; 0.01]	3.7%
Jauhiainen, T. 2006	33	-0.13	0.0200	34	-0.10	0.0300	-0.03	[-0.04; -0.02]	6.9%
Hernandez-Mijares, Antonio 2010	31	-0.07	0.1380	24	-0.01	0.1570	-0.06	[-0.14; 0.02]	3.7%
De Jong, A. 2008	15	-0.04	0.1200	11	-0.01	0.0700	-0.03	[-0.10; 0.04]	3.9%
Banuls, Celia 2010	20	-0.03	0.1250	20	-0.03	0.1650	0.00	[-0.09; 0.09]	3.2%
Takeshita, Masao 2008	14	-0.08	0.1752	15	0.01	0.2921	-0.09	[-0.26; 0.08]	1.3%
Sialvera, T. E. 2012	53	-0.08	0.2400	55	0.00	0.2300	-0.08	[-0.17; 0.01]	3.3%
Plat, J. 2009	9	-0.07	0.1000	9	-0.02	0.0900	-0.05	[-0.14; 0.04]	3.3%
Devaraj, S. 2006	36	-0.10	0.2430	36	-0.01	0.2128	-0.09	[-0.20; 0.02]	2.7%
**Random effects model**	**240**			**230**			**-0.03**	**[-0.04; -0.02]**	**33.1%**
Heterogeneity: $I^2 = 0\%$, $\tau^2 = 0$, $p = 0.85$									
**Random effects model**	**810**			**797**			**-0.04**	**[-0.06; -0.02]**	**100.0%**
Heterogeneity: $I^2 = 90\%$, $\tau^2 = 0.0018$, $p < 0.01$									
Test for subgroup differences: $\chi^2_1 = 0.01$, df = 1 ($p = 0.92$)									

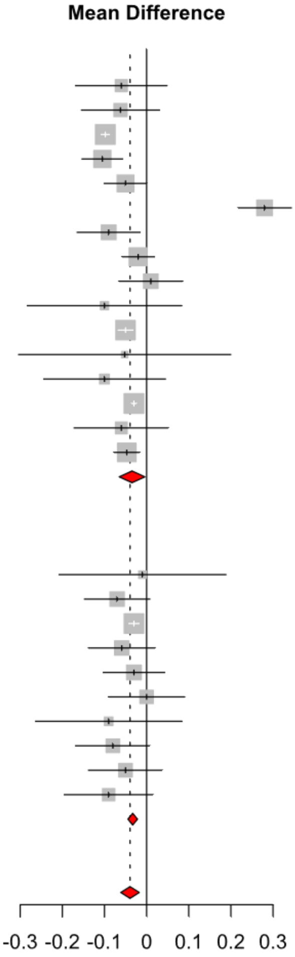

Supplement: Supplementary file 1 [file medi-103-e40020-s001.pdf]
